# Identification and validation of mitophagy-related genes in acute myocardial infarction and ischemic cardiomyopathy and study of immune mechanisms across different risk groups

**DOI:** 10.3389/fimmu.2025.1486961

**Published:** 2025-03-06

**Authors:** Ying Hao, RuiLin Li, ChengHui Fan, Yang Gao, Xia Hou, Wei wen, YunLi Shen

**Affiliations:** ^1^ Department of Cardiovascular Medicine, State Key Laboratory of Cardiovascular Diseases and Medical Innovation Center, Shanghai East Hospital, School of Medicine, Tongji University, Shanghai, China; ^2^ Department of Cardiovascular Medicine, Shanghai East Hospital Ji’an Hospital, Ji’an, Jiangxi, China

**Keywords:** mitophagy, acute myocardial infarction, ischemic cardiomyopathy, machine learning, diagnostic model

## Abstract

**Introduction:**

Acute myocardial infarction (AMI) is a critical condition that can lead to ischemic cardiomyopathy (ICM), a subsequent heart failure state characterized by compromised cardiac function.

**Methods:**

This study investigates the role of mitophagy in the transition from AMI to ICM. We analyzed AMI and ICM datasets from GEO, identifying mitophagy-related differentially expressed genes (MRDEGs) through databases like GeneCards and Molecular Signatures Database, followed by functional enrichment and Protein-Protein Interaction analyses. Logistic regression, Support Vector Machine, and LASSO (Least Absolute Shrinkage and Selection Operator) were employed to pinpoint key MRDEGs and develop diagnostic models, with risk stratification performed using LASSO scores. Subgroup analyses included functional enrichment and immune infiltration analysis, along with protein domain predictions and the integration of regulatory networks involving Transcription Factors, miRNAs, and RNA-Binding Proteins, leading to drug target identification.

**Results:**

The TGFβ pathway showed significant differences between high- and low-risk groups in AMI and ICM. Notably, in the AMI low-risk group, MRDEGs correlated positively with activated CD4+ T cells and negatively with Type 17 T helper cells, while in the AMI high-risk group, RPS11 showed a positive correlation with natural killer cells. In ICM, MRPS5 demonstrated a negative correlation with activated CD4+ T cells in the low-risk group and with memory B cells, mast cells, and dendritic cells in the high-risk group. The diagnostic accuracy of RPS11 was validated with an area under the curve (AUC) of 0.794 across diverse experimental approaches including blood samples, animal models, and myocardial hypoxia/reoxygenation models.

**Conclusions:**

This study underscores the critical role of mitophagy in the transition from AMI to ICM, highlighting RPS11 as a highly significant biomarker with promising diagnostic potential and therapeutic implications.

## 1 Introduction

Acute Myocardial Infarction (AMI), a severe form of coronary heart disease, results from sudden coronary artery occlusion, leading to myocardial ischemia and necrosis (1). Annually, it accounts for about 7 million new cases and roughly half of cardiovascular deaths globally. Ischemic Cardiomyopathy (ICM), often following AMI or reflecting advanced coronary disease, involves myocardial fibrosis from prolonged ischemia, severely affecting heart function and causing about 70% of heart failure cases (2).

Despite improved AMI management increasing survival, ICM’s prevalence is rising. In Western countries, ICM’s one-year mortality rate is around 16%, with a five-year rate near 40%. Outcomes for AMI-induced ICM patients are generally worse, with an increased risk of severe cardiac events, compared to those with non-ischemic cardiomyopathy (3).To improve the treatment of AMI and reduce the incidence of subsequent ICM, it is crucial to explore the pathophysiological mechanisms of post-myocardial infarction, identify novel biomarkers for risk stratification, recognize high-risk patients, and discover potential therapeutic targets.

Mitochondria play a pivotal role in several cellular processes including signal transduction, redox balance, and energy conversion. Cardiomyocytes, which are among the cells with the highest mitochondrial content, can undergo mitophagy in response to various stressors such as nutrient deficiency, hypoxia, DNA damage, inflammation, or mitochondrial membrane depolarization (4, 5). This process selectively removes damaged mitochondria to maintain cellular homeostasis (6, 7). During ischemia-reperfusion (I/R) injury, mitophagy is beneficial as it clears defective mitochondria. Evidence indicates that mice deficient in Drp1(dynamin - related protein 1) or Parkin manifest impaired mitophagy and exhibit an enlarged myocardial infarction area subsequent to I/R injury (8, 9). Conversely, stress-induced activation of mitophagy can lead to excessive clearance of mitochondria, resulting in inadequate ATP (Adenosine Triphosphate) synthesis and ultimately precipitating cardiomyocyte apoptosis. In experimental models, inhibition of mitochondrial fission and mitophagy by knocking down Drp1 or Mff (mitochondrial fission factor) has led to dilated cardiomyopathy (10, 11). These findings highlight the necessity of mitophagy for normal heart function and suggest that excessive mitochondrial division may be detrimental to cardiac health. The pathophysiological mechanisms of mitophagy in AMI and ICM are still unclear, and it remains uncertain whether the extent of mitophagy affects the prognosis of these diseases. Further investigation of its regulatory mechanisms is of significant importance for the treatment of these diseases.

Machine learning algorithms are increasingly employed in bioinformatics analysis, capable of managing dynamic, voluminous, and complex datasets. These algorithms can detect trends and patterns potentially overlooked by human analysis, thereby significantly enhancing the reliability of diagnostic systems. Previous studies have applied machine learning to analyze and identify mechanisms and biomarkers for the development of ischemic heart failure following acute myocardial infarction (12). However, these studies often provide broad conclusions and do not specifically address mitophagy. In research conducted by ZhiKai Yang and colleagues, various machine learning algorithms were utilized to study differences in mitophagy between patient groups with acute myocardial infarction and stable coronary artery disease (13). While this research underscored the significant role of mitophagy in coronary artery disease, it did not address the subset of patients with the worst prognosis who progress from myocardial infarction to ischemic cardiomyopathy.

**Figure 1:**
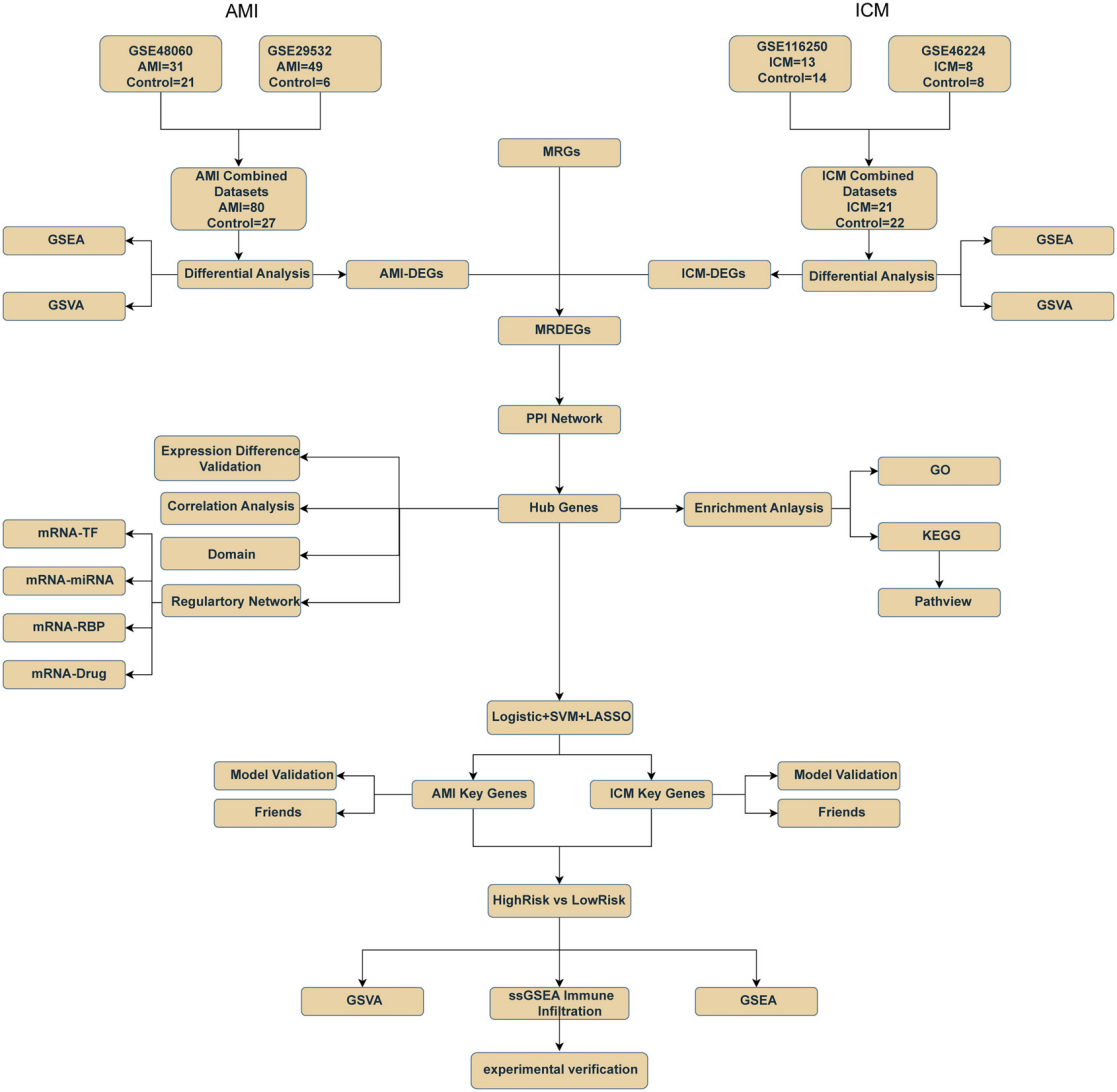
Flow chart for the comprehensive analysis of MRDEGs. AMI, Acute Myocardial Infarction; ICM, Ischemic Cardiomyopathy; DEGs, Differentially Expressed Genes; MRGs, Mitophagy-Related Genes. MRDEGs, Mitophagy-Related Differentially Expressed Genes; SVM, Support Vector Machines; LASSO, Least Absolute Shrinkage and Selection Operator; ROC, Receiver Operating Characteristic; GSEA, Gene Set Enrichment Analysis; GSVA, Gene Set Variation Analysis, PPI Network, Protein-Protein Interaction Network. GO, Gene Ontology; KEGG, Kyoto Encyclopedia of Genes and Genomes; TF, Transcription Factor; RBP, RNA-Binding Protein.

This study conceptualized AMI and ICM as stages of a single pathological process, using bioinformatics and machine learning to explore mitophagy’s role (**Figure 1**). We identified key mitophagy genes and signaling pathways influencing the transition from AMI to ICM, revealing potential biomarkers for diagnosis, risk stratification, and new insights into the treatment and prognosis of these cardiovascular conditions.

## 2 Materials and methods

### 2.1 Data collection and processing

Using the R package GEOquery (14), we downloaded two datasets each for AMI [GSE48060 (15) and GSE29532 (16)] and ICM [GSE116250 (17) and GSE46224 (18)] from the GEO (19) database (https://www.ncbi.nlm.nih.gov/geo/). Comprehensive details are available in **Supplementary Tables S1**, **S2**. The R package sva (20) was utilized for batch correction and integration, producing the consolidated GEO datasets for AMI and ICM. The R package limma (21) facilitated normalization and standardization, followed by principal component analysis (22). Mitophagy-related genes (MRGs) were sourced from the GeneCards database (23) (https://www.genecards.org/) and the Molecular Signatures Database (MSigDB) (24) (https://www.gsea-msigdb.org/gsea/msigdb), yielding a total of 1633 unique MRGs (mitophagy-related genes) after merging and deduplication, as detailed in **Supplementary Table S3**.

### 2.2 Differentially expressed genes between AMI and ICM

The analysis of differential gene expression was carried out for both AMI and ICM using the limma package in R. After reviewing literature (25, 26) and testing various thresholds, we chose |logFC| > 0 and P < 0.05 to ensure robust results while maximizing the inclusion of as many biologically significant differentially expressed genes as possible. Genes with logFC above 0 and a p-value below 0.05 were categorized as up-regulated, whereas those with logFC below 0 and the same p-value threshold were categorized as down-regulated. Venn diagrams were employed to depict the overlap between up-regulated and down-regulated genes, and further intersections with MRGs (mitophagy-related genes) were analyzed to pinpoint MRDEGs (mitophagy-related differentially expressed genes).

### 2.3 Protein-protein interaction network construction and hub gene selection

The STRING database (27) (https://string-db.org/) facilitated the construction of a PPI network based on MRDEGs(mitophagy-related differentially expressed genes), employing a minimum interaction confidence score of 0.400(medium confidence). Interactions with a confidence score above this threshold are considered to be sufficiently supported by evidence, thereby filtering out potential false-positive results. The CytoHubba (28) plugin within Cytoscape (29) software applied five algorithms—Maximum Neighborhood Component (MNC), Maximal Clique Centrality (MCC), Edge Percolated Component (EPC), Degree, Closeness (30)—to compute scores for MRDEGs, selecting the top 20 MRDEGs. The intersection of results from these algorithms identified hub genes related to AMI and ICM. By performing multi-analysis screening using the STRING database and five algorithms in Cytoscape, the reliability of the results was enhanced, and errors that might arise from relying on a single algorithm were minimized.

### 2.4 Protein domain prediction and regulatory network construction

AlphaFoldDB (31) (https://alphafold.com) predicted and visually displayed the protein structures of hub genes, assessed by a Predicted Local Distance Difference Test (pLDDT) score ranging from 0 to 100. The regulatory network between the mRNA of 9 hub genes and 48 transcription factors (TFs) was predicted using the ChIPBase (32) database (http://rna.sysu.edu.cn/chipbase/). Potential interactions between mRNA and miRNAs, as well as mRNA and RNA-binding proteins (RBPs) (33), were screened using the StarBase v3.0 database (34) (https://starbase.sysu.edu.cn/), and the networks were visualized using Cytoscape software. This analysis included 4 hub genes and 27 miRNAs, as well as 10 hub genes and 43 RBPs. Furthermore, the Comparative Toxicogenomics Database(CTD) (35) (https://ctdbase.org/) was employed to identify potential drugs or molecular compounds associated with the hub genes. The mRNA-Drug regulatory network was constructed and subsequently visualized using Cytoscape software, comprising 8 hub genes and 15 drugs or molecular proteins.

### 2.5 Hub gene expression difference and correlation analysis

Expression levels of MRDEGs(mitophagy-related differentially expressed genes) in the Combined Datasets were compared using group comparison graphs. The Spearman algorithm analyzed the correlation of hub gene expressions, with the R packages igraph (36) and ggraph illustrating correlations and chord diagrams. Scatter plots by the ggplot2 R package displayed the strongest correlated hub genes.

### 2.6 Functional enrichment analysis of MRDEGs

Gene Ontology(GO) (37) and Kyoto Encyclopedia of Genes and Genomes(KEGG) (38) enrichment analysis of hub genes was performed using the R package clusterProfiler (39), electing results based on an adjusted p-value < 0.05. The Pathview R package (40) visualized the pathway enrichment analysis results.

### 2.7 GSEA and GSVA analysis

GSEA (41) (Gene Set Enrichment Analysis)analysis was executed on the combined datasets for AMI and ICM using the clusterProfiler package in R, with the following settings: seed value at 2023, a gene set size range from 10 to 500, and the gene set c2.cp.all.v2022.1.Hs.symbols.gmt [All Canonical Pathways]. The threshold for significance was set at a p-value below 0.05. Additionally, GSVA (42) (Gene Set Variation Analysis)was applied to all genes within the combined datasets of AMI and ICM, utilizing gene sets from MSigDB (24), adhering to the same p-value criterion for selection.

### 2.8 Diagnostic model construction

We utilized multiple machine learning algorithms, including Logistic Regression, Support Vector Machine (SVM) (43), and Least Absolute Shrinkage and Selection Operator (LASSO) regression analysis, to identify key genes for constructing diagnostic models for AMI and ICM. This approach is grounded in several studies of significant scientific value in the field of bioinformatics (44, 45). The models were implemented using the R package glmnet, with parameters set.seed (500) and family=‘binomial’ (46).The key genes chosen from AMI and ICM to determine the RiskScore, employing coefficients obtained from LASSO regression analysis.


$$RiskScore\;=\;\sum_{i}Coefficient\;\left(gene_{i}\right)*mRNA\;Expression\;\left(gene_{i}\right)$$

### 2.9 Diagnostic model validation and key gene ROC curve analysis

ROC(Receiver Operating Characteristic) curves were plotted for the diagnostic models of AMI Key Genes and ICM Key Genes using the pROC package in R. Additionally, nomograms (47) illustrating the relationships between Key Genes were generated with the rms package in R. Calibration analysis was conducted to evaluate the precision and discriminatory capacity of the diagnostic models for AMI and ICM. Decision Curve Analysis (DCA) (48) for predicting clinical outcomes using AMI Key Genes and ICM Key Genes was performed using the ggDCA package in R. Moreover, Functional Similarity (Friends) analysis was carried out with the GOSemSim R package (49).

### 2.10 High- and low-risk group differential expression analysis, GSEA, GSVA

To enhance the reliability of our methodology, we drew upon the approach proposed by Zhang L et al. (50), and utilized mitophagy-related RiskScore to subgroup the AMI group for further in-depth analysis. Based on the formula outlined in section 2.8, we calculated the RiskScore for acute myocardial infarction (AMI) samples within the AMI Combined Datasets, utilizing the regression coefficients derived from the LASSO model specifically for AMI. The median RiskScore was instrumental in categorizing the samples into HighRisk and LowRisk groups. Samples with a risk score above the median were classified into the HighRisk group, while those with a risk score equal to or below the median were classified into the LowRisk group. A similar methodology was applied to determine the RiskScore for ischemic cardiomyopathy (ICM) samples in the ICM Combined Datasets, again using the LASSO regression coefficients pertinent to ICM. The samples were classified into HighRisk and LowRisk categories based on their median RiskScores. These two sets of high- and low-risk classifications will be utilized for subsequent subgroup analyses independently. Differential analysis was carried out with the limma package in R, with visualization of the results achieved through the ggplot2 and pheatmap packages in R.

GSEA (41) was conducted on AMI samples in the AMI Combined Datasets and ICM samples in the ICM Combined Datasets with clusterProfiler package in R. GSVA (42) was applied to the HighRisk and LowRisk groups of AMI and ICM samples, respectively. The same gene sets, parameters, and screening criteria were used as in previous analyses.

### 2.11 Immune infiltration analysis of HighRisk and LowRisk groups

Immune cell infiltration matrices were determined through single sample gene set enrichment analysis (ssGSEA) (51) for samples of AMI and ICM. Comparison graphs for the groups were created using ggplot2 to illustrate the variance in immune cell expression between the LowRisk and HighRisk groups in AMI and ICM. The detailed subgroup classification method can be found in section 2.10.

### 2.12 Validation of peripheral blood samples

The Ethics Committee of Shanghai East Hospital, affiliated with Tongji University, approved this study(Approval number 2024-175), which follows the Declaration of Helsinki guidelines. Once written informed consent was secured from all participants, peripheral blood samples were collected from six individuals each with diagnoses of AMI and ICM, and from six normal subjects.

Venous blood samples were collected into whole blood RNA preservation tubes (model ZXQX-10). After centrifugation and sedimentation, total RNA was extracted from peripheral blood mononuclear cells (PBMCs) employing the TransZol Up Plus RNA kit (TransGen, China). The integrity and purity of the extracted RNA were evaluated using the GEN5 microplate reader (biotek, USA). Quantitative real-time PCR (RT-qPCR) experiments were performed on the Q5 Real-Time PCR Detection System (Thermo, USA). Glyceraldehyde-3-phosphate dehydrogenase (GADPH) was used as the internal control to normalize the data. Relative expression levels of the target genes were determined using the 2-ΔΔCt method.

### 2.13 Experimental validation

#### 2.13.1 Experimental animals

For the animal experiments, we obtained eight-week-old male C57BL/6 mice from Shanghai Lingchang Biotechnology Co. (Shanghai, China). The mice were housed under controlled conditions of temperature (23°C) and humidity (65%) with a 12/12-hour light/dark cycle. All experimental procedures were conducted in strict compliance with national regulations regarding animal welfare and ethics. The study was approved by the Ethics Committee of Shanghai East Hospital, associated with Tongji University.

#### 2.13.2 Establishment of myocardial infarction model

Twenty-four mice were randomly assigned into two groups: Myocardial Infarction (MI) and Sham, with 12 mice in each group. In each group, six mice were randomly selected for histological staining and immunohistochemistry, while the remaining six were used for molecular analyses. Myocardial infarction was induced in the MI group by ligating the left anterior descending (LAD) coronary artery. Post-ligation, the myocardium exhibited a color change from bright red to pale, accompanied by a gradual weakening of contraction. Electrocardiographic (ECG) monitoring confirmed the successful establishment of the MI model, as indicated by ST-segment elevation and the presence of a J wave following the ST-segment. The Sham group underwent the same surgical procedure without LAD ligation. Cardiac function was assessed via echocardiography on the day following surgery.

#### 2.13.3 Hematoxylin & eosin, masson staining, and immunohistochemistry

Hematoxylin and Eosin (HE) staining and Masson staining were performed on cardiac tissue sections using the respective kits (Beyotime, C0105M). These staining procedures were used to observe and analyze the morphological characteristics of the cardiac tissues. The heart tissue sections were deparaffinized, rehydrated, autoclaved with citrate buffer (pH 6.0) for 10 minutes for antigen repair, cooled to room temperature, and then sealed for 15 minutes with 3% H_2_O_2_ for endogenous peroxidase activity. Sections were washed with PBS (Phosphate - Buffered Saline) and blocked with 10% goat serum for 30 minutes. They were then incubated overnight at 4°C with a primary antibody (anti-*RPS11* antibody, 1:200, Proteintech, 17041-1AP). The following day, biotinylated secondary antibody (1:500) and streptavidin-HRP(Horseradish Peroxidase) were incubated sequentially at room temperature for 30 minutes each after washing with PBS, and the nuclei were washed with PBS and stained with DAB(3,3’ - Diaminobenzidine tetrahydrochloride), and the nuclei were lightly post-stained with hematoxylin. The expression of *RPS11* protein was indicated by a brownish-yellow signal, and the area and intensity of the positive signal were analyzed using Image-Pro Plus software.

#### 2.13.4 RT-PCR

Total RNA was extracted from mouse heart tissues using Trizol reagent (Beyotime, R0016). The detailed methods, steps, and reagents follow those described in section 2.12, RT-PCR operations.

#### 2.13.5 Establishment of the myocardial H/R model and detection of apoptosis rate by flow cytometry

In this study, H9c2 cardiomyocytes were selected and cultured at 37°C and 5% CO_2_ in sugar-rich DMEM(Dulbecco’s Modified Eagle Medium) medium supplemented with 10% fetal bovine serum (FBS) and 1% penicillin/streptomycin (P/S). When the cells reached the logarithmic phase, they were divided into two groups according to the experimental requirements: the normoxic control group (the Control group) and the hypoxia-reoxygenation group (the H/R group). In the construction of the hypoxia-reoxygenation model, the cells in the hypoxia-reoxygenation group(the H/R group) were first placed in a three-gas incubator (1% O_2_, 5% CO_2_, 94% N_2_) with sugar-free DMEM instead of the conventional medium for a 4-hour hypoxia treatment; then the cells were replaced with the conventional medium (sugar-rich DMEM, 10% FBS, 1% P/S) and placed in the conventional medium at 37°C and 5% CO_2_ for a 4-hour reoxygenation. The cells of the normoxic control group (the Control group) were always cultivated in a conventional incubator without changing the culture medium. After the establishment of the model, the cells in each group were subjected to flow cytometry using the Annexin V-FITC/PI double staining kit (MCE,HY-K1073), and the apoptosis rate was analyzed according to the instructions of the kit.

#### 2.13.6 Gene knockdown via plasmid transfection & western blot

To investigate the role of the *RPS11* gene in the hypoxia/reoxygenation process of cardiomyocytes, H9c2 cardiomyocytes were divided into three groups in this experiment: Hypoxia-reoxygenation group (the H/R group), hypoxia-reoxygenation + *RPS11* knockdown group (the H/R+si*RPS11* group) and hypoxia-reoxygenation + empty vector control group (the H/R+siCON group). Knockdown transfection was performed 24 hours before hypoxia treatment with siRNA Transfection Reagent (Sigma-Aldrich, SITRAN-RO) according to the instructions. The cells in the H/R+si*RPS11* group were transfected with the si*RPS11* plasmid (MCE, HY-RS12221); the cells in the H/R+siCON group were transfected with the empty plasmid (siCON). Twenty-four hours after transfection, the cells were placed in a triple gas incubator for a 4-hour hypoxia treatment (1% O_2_, 5% CO_2_, 94% N_2_). The cells were shifted to a regular incubator following the replacement of the standard medium for a 4-hour reoxygenation phase at 37°C and no CO_2_. The anoxia treatment and reoxygenation methods were performed as described previously.

After modeling each experimental group, cellular protein samples were collected for Western blot assay. After the protein samples were lysed with RIPA (Radioimmunoprecipitation Assay Buffer) lysate, the total protein concentration was determined using the BCA (Bicinchoninic Acid) protein concentration assay kit (Thermo Fisher Scientific, USA), and the same amount of protein (30µg per well) was loaded onto an SDS-PAGE (Sodium Dodecyl Sulfate - Polyacrylamide Gel Electrophoresis) gel for electrophoresis. After electrophoresis, proteins were transferred to PVDF (Polyvinylidene Fluoride) membranes (Millipore, USA), sealed with 5% skimmed milk powder for 1 hour at room temperature, and then incubated with primary antibodies (including anti-GAPDH (MCE, HY-P80137), anti-β-actin (MCE, HY-P80438), anti-RPS11 (Proteintech, 17041-1AP), anti-BNIP3(BCL2 protein-interacting protein 3) (MCE, HY-P80035), and anti-LC3II/I (Microtubule-associated protein 1 light chain 3 II/I) (Aladdin,Ab112877) separately at 4°C overnight. The membranes were treated as follows the next day: they were washed three times, for 10 minutes each, with PBST(Phosphate - Buffered Saline Tween), and then incubated for an hour with the secondary antibodies (HRP-marked). Protein signals were detected using the ECL(Enhanced Chemiluminescence) chemiluminescence kit (MACKLIN, E917966), and the grey levels of target proteins were analyzed using Image Lab software (Bio-Rad, USA) and normalized using GAPDH and β-actin as internal references. The experiment was repeated three times and the results were expressed as mean ± standard deviation.

### 2.14 Statistical analysis

This article’s data processing and analyses were performed with R software (Version 4.3.0). We assessed the statistical significance of continuous variables across two groups using the independent Student’s T-Test. For variables that did not follow a normal distribution, the Mann-Whitney U test (also referred to as the Wilcoxon Rank Sum Test) was utilized. The Kruskal-Wallis test was applied to analyze data involving three or more groups. Spearman’s correlation analysis determined the relationships among various molecules. All statistical tests were two-sided, and a p-value threshold of less than 0.05 was considered significant.

## 3 Results

### 3.1 Identification of differentially expressed genes

**Figure 2:**
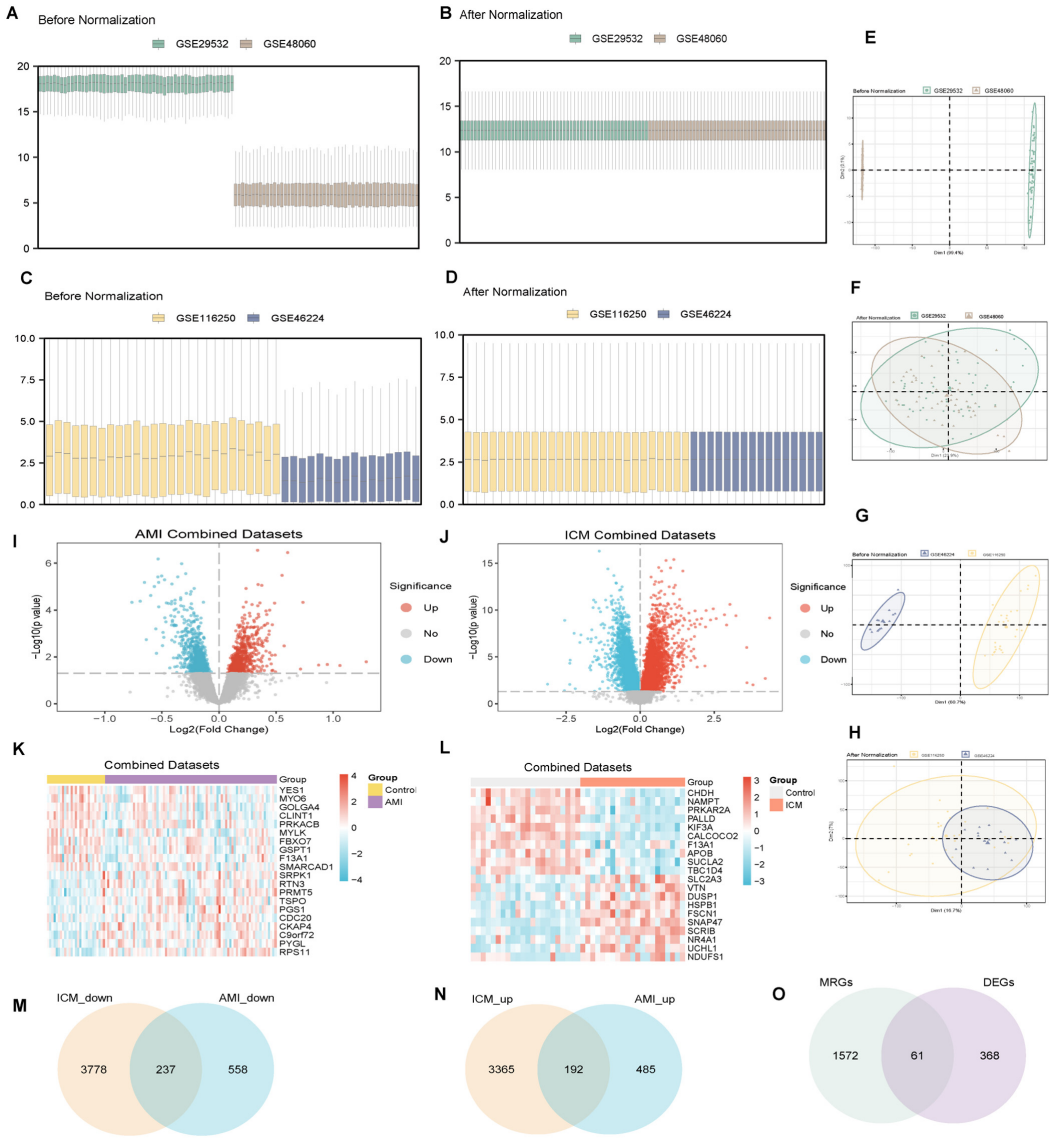
Data set standardization, differential gene expression analysis. **(A-H)** Standardization and batch removal of AMI **(A, B, E, F)** and ICM **(C, D, G, H)** combined datasets. **(I-O)** Differential gene expression analysis for AMI **(I, K)** and ICM **(J, L)**, with Venn diagrams illustrating down-regulated **(M)** and up-regulated genes **(N)**, and common DEGs and MRGs **(O)**.

Batch effects were meticulously removed from AMI datasets GSE48060 and GSE29532, culminating in the creation of the AMI Combined Datasets (**Figures 2A, B, E, F**). In a similar manner, batch effects were eliminated from RPKM(Reads Per Kilobase of transcript per Million mapped reads)data for ICM datasets GSE116250 and GSE46224, producing the ICM Combined Datasets (**Figures 2C, D, G, H**).

Within the AMI Combined Datasets, a total of 1472 genes were identified as differentially expressed, encompassing 677 upregulated and 795 downregulated genes (**Figures 2I, K**). Concurrently, the ICM Combined Datasets revealed 7572 DEGs(differentially expressed genes), including 3557 upregulated and 4015 downregulated genes (**Figures 2J, L**).

A comparison of genes from both datasets showed that 429 genes were differentially expressed in both AMI and ICM, with 237 downregulated (**Figure 2M**) and 192 of these genes upregulated (**Figure 2N**). Additionally, 61 MRDEGs(mitophagy-related differentially expressed genes) were isolated through the intersection of the DEGs with genes related to mitophagy (**Figure 2O**), detailed further in **Supplementary Table S4**.

### 3.2 Building the PPI network and determining hub genes

**Figure 3:**
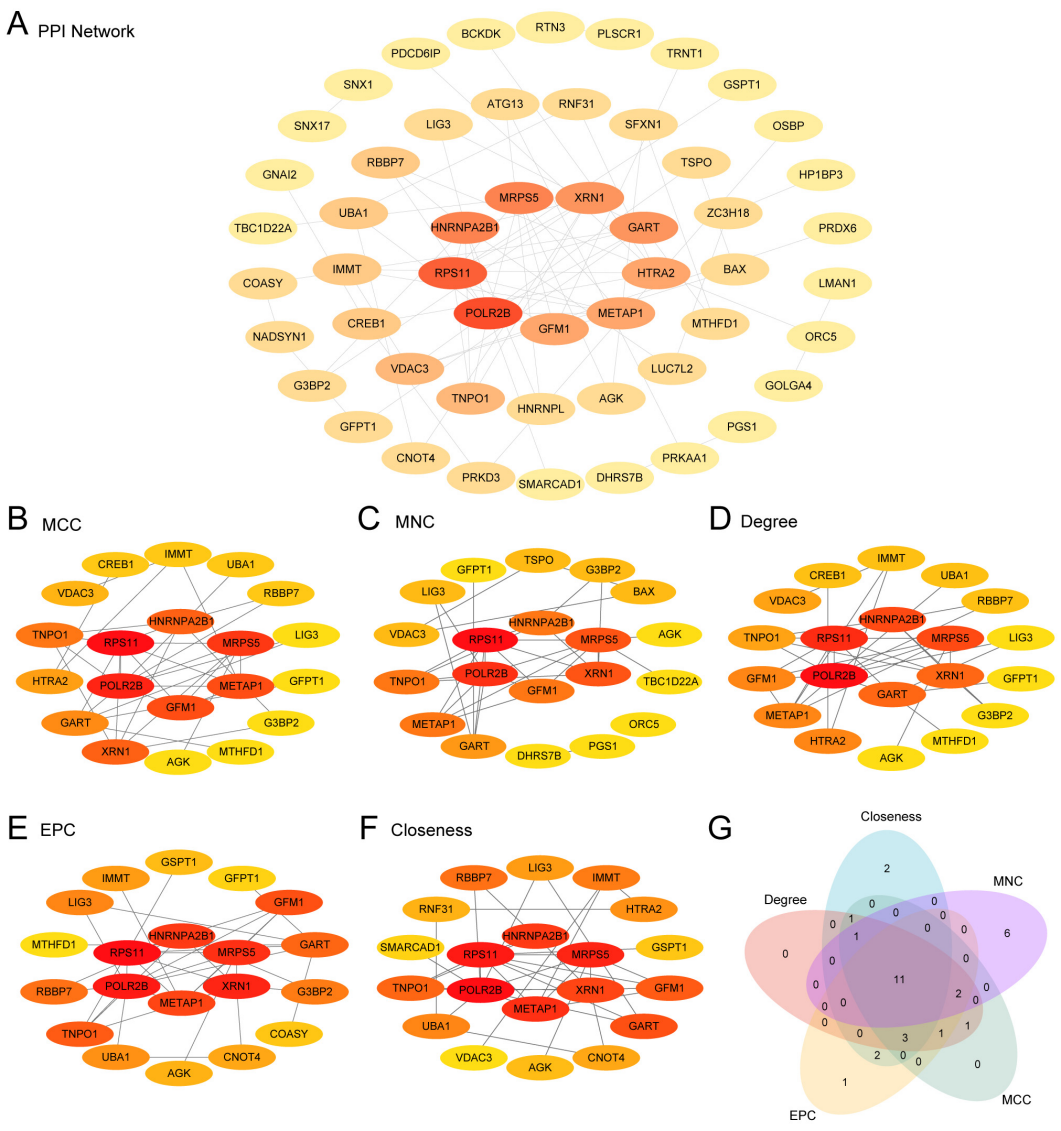
PPI network and hub genes analysis. **(A)** PPI network from the STRING database of mitophagy-related differentially expressed genes (MRDEGs) calculated from STRING database. **(B-F)** PPI networks of the top 20 MRDEGs identified by five CytoHubba algorithms [MCC (Maximal Clique Centrality), MNC (Maximum Neighborhood Component), Degree, Closeness, EPC (Edge Percolated Component)]. **(G)** A Venn diagram of the top 20 MRDEGs.

Utilizing the STRING database, an interrelationship was established among 52 MRDEGs (mitophagy-related differentially expressed genes), forming a robust PPI network (**Figure 3A**). These genes were evaluated and ranked using five distinct algorithms within Cytoscape, leading to the identification of the top 20 MRDEGs (**Figures 3B–F**). A cross-analytical approach among these algorithms revealed 11 critical Hub Genes associated with both AMI and ICM: *POLR2B*, *RPS11, MRPS5, METAP1, HNRNPA2B1, XRN1, GART, GFM1, TNPO1, LIG3*, and *AGK* (**Figure 3G**).

### 3.3 Protein domain prediction and regulatory network construction

**Figure 4:**
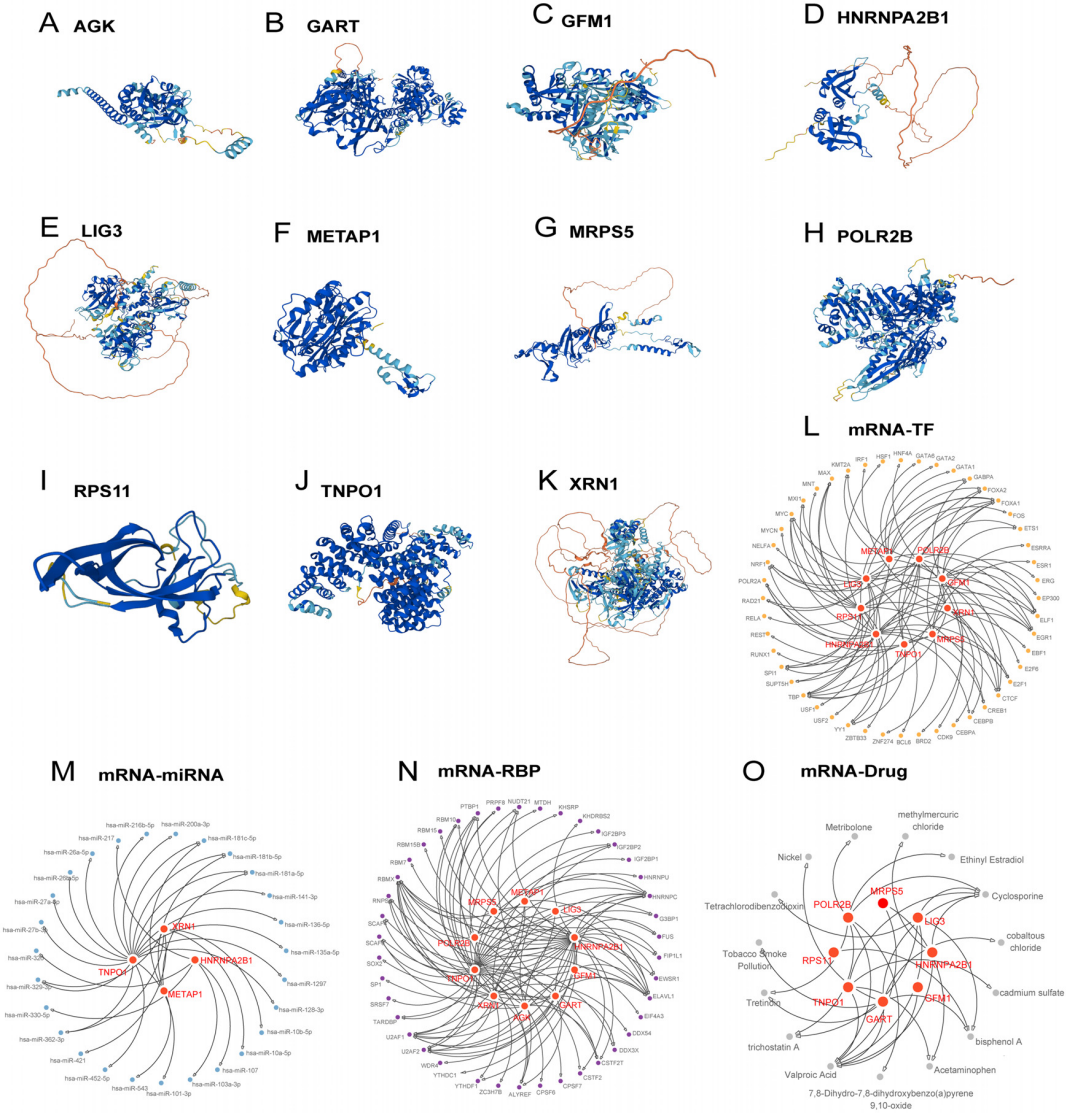
Protein structure, regulatory network of hub genes. **(A-K)** Protein structures of hub genes. **(L-O)** Regulatory networks of hub genes: mRNA-TF **(L)**, mRNA-miRNA **(M)**, mRNA-RBP **(N)**, mRNA-Drug **(O)**.

Protein structures for the 11 Hub Genes were predicted and visualized using AlphaFoldDB (**Figures 4A–K**). Nine of these genes demonstrated high structural confidence (pLDDT > 90) across their main domains: *POLR2B, RPS11, MRPS5, METAP1, HNRNPA2B1, XRN1, GART, TNPO1*, and *AGK;* the remaining two, *LIG3* and *GFM1*, showed substantial confidence (70 < pLDDT < 90).

The regulatory network was expanded to include 48 transcription factors linked to 9 Hub Genes, constructing an mRNA-TF network (**Figure 4L**, detailed in **Supplementary Table S5**). Moreover, regulatory networks involving 27 miRNAs binding to 4 hub genes and 43 RBPs associated with 10 Hub Genes were elucidated (**Supplementary Table S5**, **Figures 4M, N**, respectively). An mRNA-drug interaction network involving 8 Hub Genes and 15 drugs or molecular compounds was also constructed (**Figure 4O**, detailed in **Supplementary Table S5**).

### 3.4 Hub gene expression difference and correlation analysis

**Figure 5:**
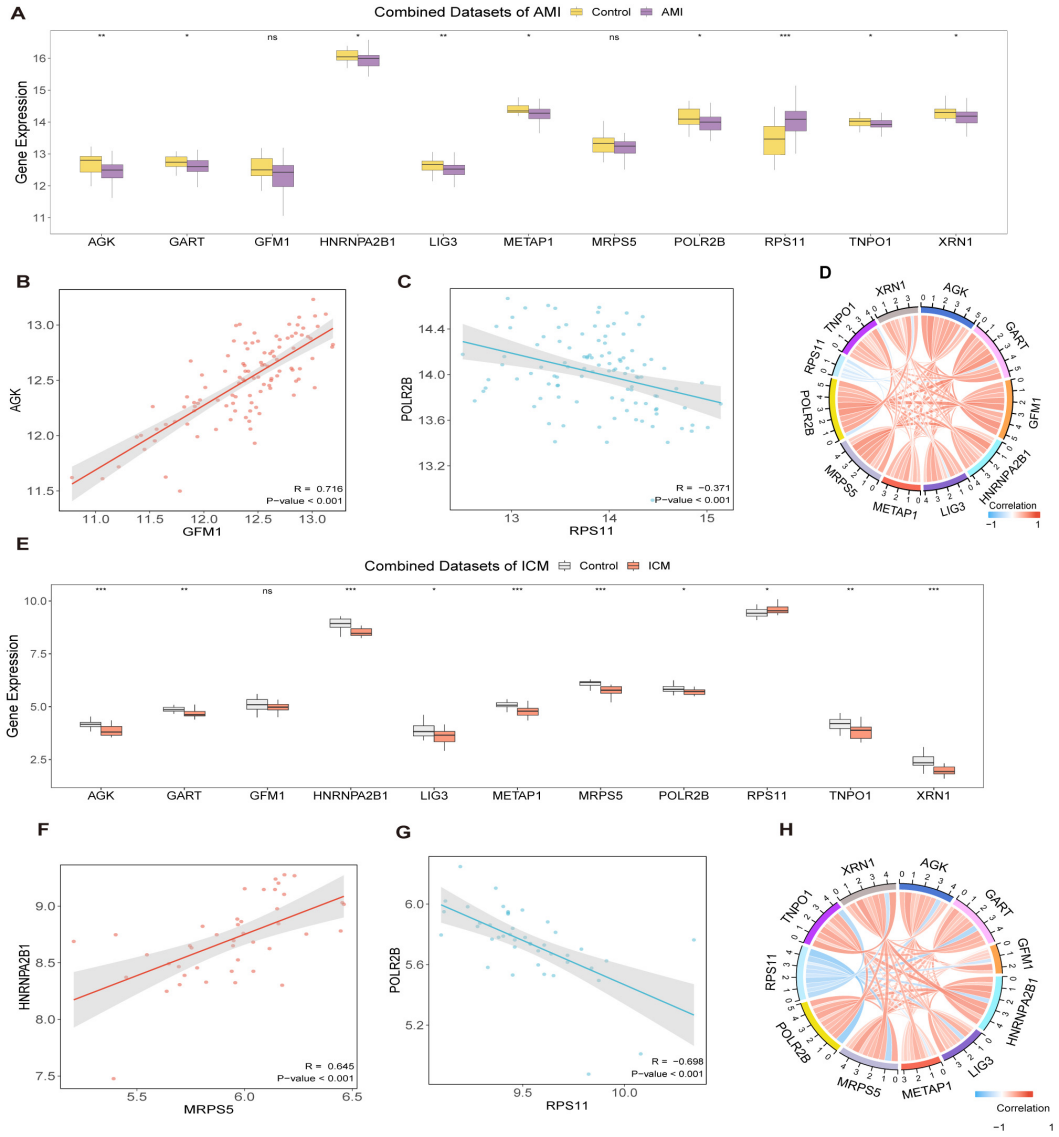
Differential expression and correlation analysis. Hub gene comparison in AMI dataset **(A)** and in ICM dataset **(E)**. Correlation and chord plots of hub genes in AMI datasets **(D)** and ICM datasets **(H)**. Correlation scatter plots between Hub Genes in AMI datasets **(B, C)** and in ICM datasets **(F, G)**. (***p < 0.001, **p < 0.01, *p < 0.05, ns p >0.05).

Substantial differences were detected in the expression levels of 9 Hub Genes between the AMI group and the control group, with genes *AGK, GART, HNRNPA2B1, LIG3, METAP1, POLR2B, RPS11, TNPO1*, and *XRN1* showing statistically significant differences (p-value < 0.05) (**Figures 5A, D**). The most pronounced positive correlation was between *GFM1* and *AGK*, showing a p-value less than 0.001 and a correlation coefficient (r-value) of 0.716 (**Figure 5B**), while the strongest negative correlation was noted between *RPS11* and *POLR2B*, with an r value of -0.371 and a p-value less <0.001 (**Figure 5C**).

When contrasting the ICM group with the control group, 10 Hub Genes revealed noteworthy distinctions in their expression levels, with statistical significance (p-value < 0.05): *AGK, GART, HNRNPA2B1, LIG3, METAP1, MRPS5, POLR2B, RPS11, TNPO1*, and *XRN1* (**Figures 5E, H**). In this group, the strongest positive correlation was found between *MRPS5* and *HNRNPA2B1* (r-value = 0.645, p-value < 0.001, **Figure 5F**), while the most significant negative correlation was noted between *RPS11* and *POLR2B* (r- value = -0.698, p-value < 0.001, **Figure 5G**).

### 3.5 Functional enrichment analysis

**Figure 6:**
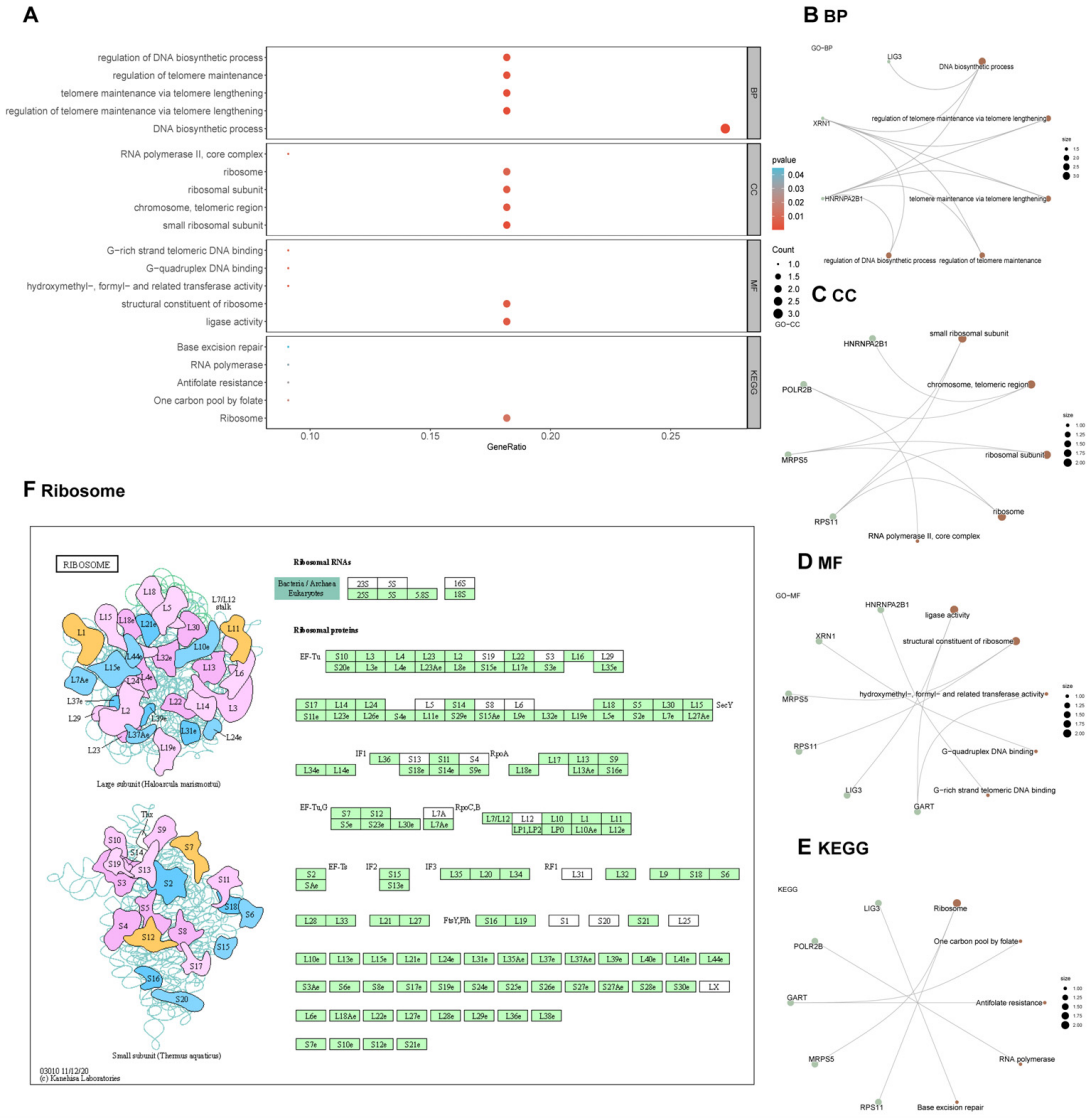
GO and KEGG enrichment analysis for hub genes. **(A-E)** GO and KEGG enrichment analysis for hub genes. **(F)** Visualization of the Ribosome pathway in the KEGG pathway enrichment analysis.

After converting the hub genes into gene IDs, comprehensive GO and KEGG analyses were performed. Key biological processes identified included the DNA biosynthetic process, the regulation of telomere maintenance via telomere lengthening, telomere maintenance via telomere lengthening, regulation of DNA biosynthetic process (**Figures 6A–D**, **Supplementary Table S6**). The KEGG pathway analysis further elucidated the relationship between Hub Genes and critical signaling pathways. These genes exhibited notable enrichment primarily in pathways such as Ribosome Pathway, the Antifolate Resistance, One Carbon Pool by Folate, RNA polymerase, and Base excision repair (**Figures 6E, F**, **Supplementary Figure 1**).

### 3.6 GSEA and GSVA

**Figure 7:**
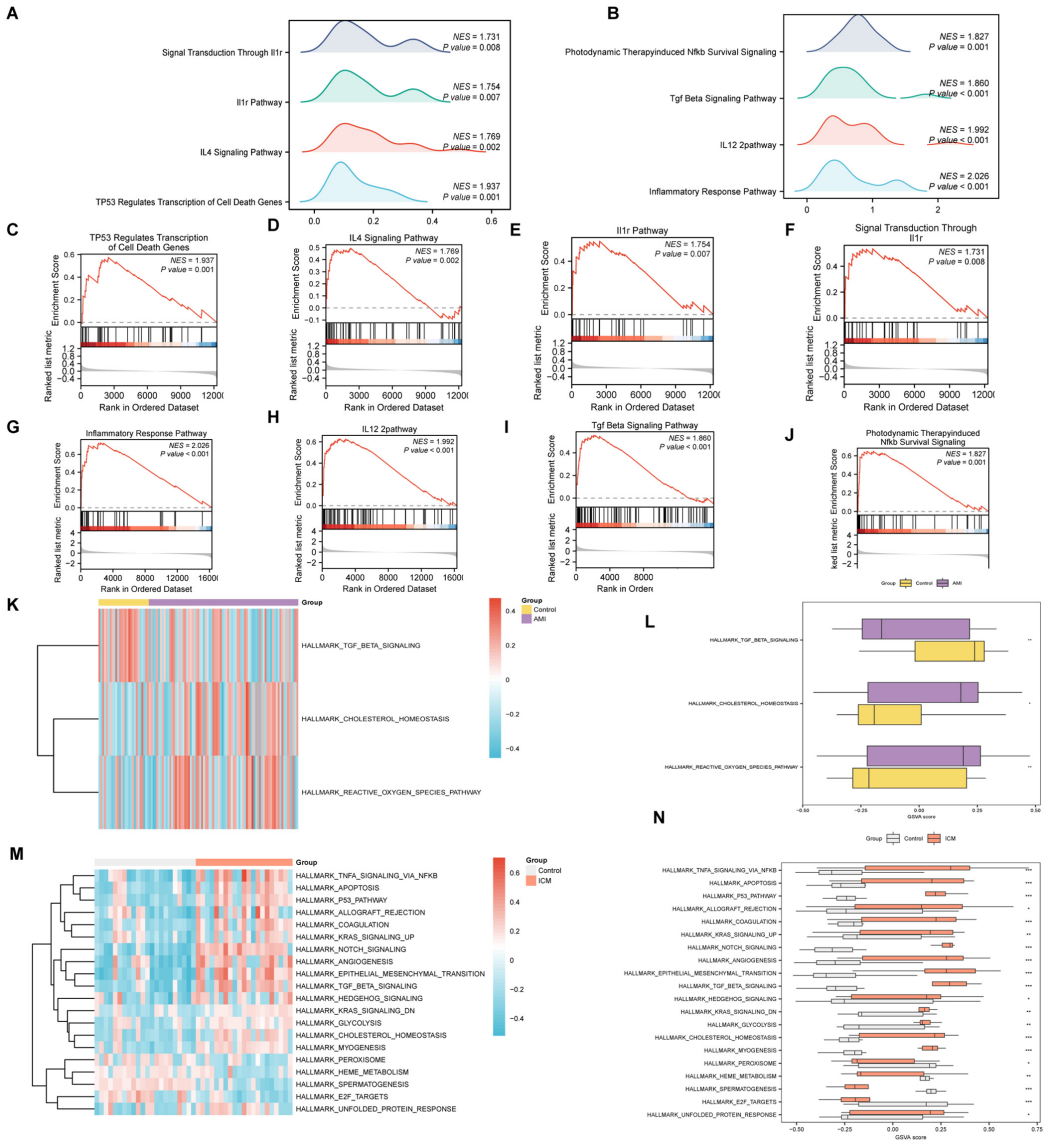
GSEA and GSVA analysis for AMI and ICM combined datasets. **(A-J)** GSEA of AMI and ICM combined datasets, detailing biological functions [**(A)** for AMI, **(B)** for ICM] and enrichment pathways [**(C-F)** for AMI, **(G-J)** for ICM]. **(K-N)** GSVA for AMI and ICM datasets, represented by heat maps [**(K)** for AMI, **(M)** for ICM] and group comparison maps [**(L)** for AMI, **(N)** for ICM]. (***p < 0.001, **p < 0.01, *p < 0.05, ns p >0.05).

The GSEA highlighted that all genes in the AMI Combined Datasets were significantly associated with biological functions including regulation of cell death, inflammatory mediators, and their signaling pathways (**Figures 7A, C–F**, **Supplementary Table S7**). Similarly, genes in the ICM Combined Datasets were predominantly linked to inflammatory responses and pathways like Tgf Beta, Nfkb, among others (**Figures 7B, G–J**, **Supplementary Table S8**).

In the GSVA, significant distinctions were noted in the enrichment of specific functions and pathways between the datasets. For the AMI Combined Datasets, notable pathways included Reactive Oxygen Species Pathway, Cholesterol Homeostasis, and Tgf Beta Signaling (**Figures 7K, L**, **Supplementary Table S9**). In the ICM Combined Datasets, numerous pathways demonstrated significant enrichment. By applying stringent criteria, including a p-value < 0.05 and ranking by logFC, we identified the top 10 pathways demonstrating positive enrichment as well as the top 10 pathways displaying negative enrichment. This selection includes pathways such as Unfolded Protein Response, E2f Targets, Spermatogenesis, Heme Metabolism, Peroxisome, and Myogenesis, among others (**Figures 7M, N**, **Supplementary Table S10**).

### 3.7 Confirmation of key genes through machine learning

**Figure 8:**
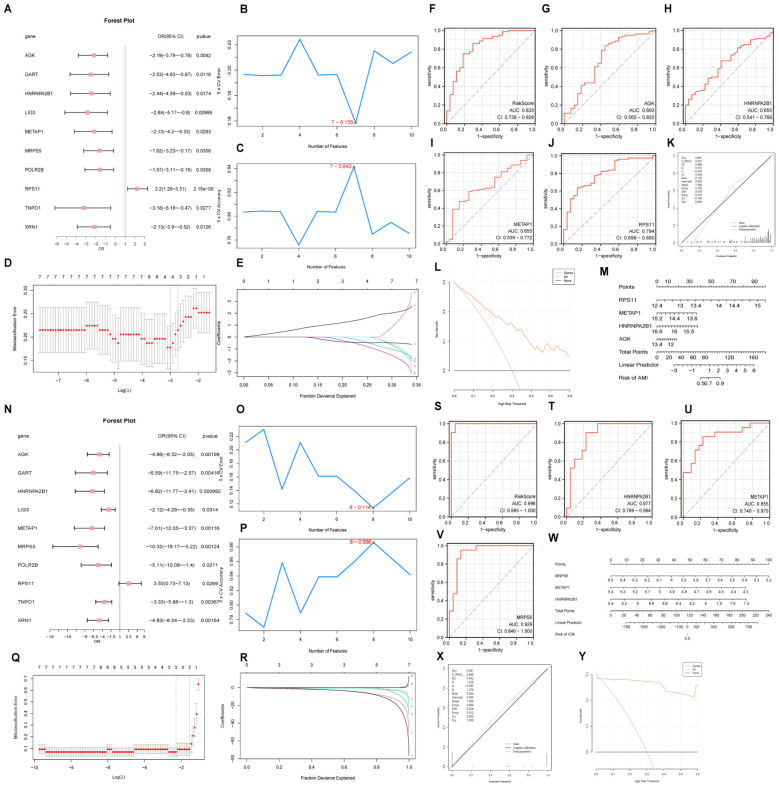
Diagnostic model and ROC curve analysis. Forest plots of hub genes included in the logistic regression model for AMI **(A)** and ICM **(N)**. Visualization of genes with the lowest error rate [**(B)** for AMI, **(O)** for ICM] and highest accuracy [**(C)** for AMI, **(P)** for ICM] obtained by the SVM algorithm. Diagrams of variable trajectories and diagnostic models using the LASSO regression model for AMI **(D, E)** and ICM **(Q, R)**. ROC curves for risk scores and key genes in AMI **(F-J)** and ICM **(S-V)** datasets. Nomograms, calibration curves, and decision curve analysis (DCA) plots for key genes in AMI **(K–M)** and ICM **(W–Y)**.

For AMI, a logistic regression model utilizing 11 hub genes identified 10 significant contributors (**Figure 8A**). Additionally, an SVM(support vector machine) model highlighted 7 pivotal genes with minimal error rates and maximum accuracy (**Figures 8B, C**). Critical genes such as *RPS11* and *AGK* were further affirmed through LASSO(Least Absolute Shrinkage and Selection Operator) regression, which refined the AMI diagnostic model to include 4 key genes: *RPS11*, *METAP1, HNRNPA2B1*, and *AGK* (**Figures 8D, E**). In ICM diagnosis, logistic regression, SVM, and LASSO models emphasized the diagnostic relevance of genes like *MRPS5, METAP1*, and *HNRNPA2B1* (**Figures 8N–R**).

### 3.8 Diagnostic value assessment

The ROC(Receiver Operating Characteristic) curve generated from the RiskScore of the diagnostic model for AMI demonstrated an AUC (Area Under the Curve) of 0.833 (**Figure 8F**), indicating high diagnostic accuracy. The ROC curve for the key gene *RPS11* had an AUC value of 0.794, with other genes having AUC values between 0.5 and 0.7 (**Figures 8G–J**). The nomogram highlighted the significant contribution of *RPS11* expression to improving the diagnostic utility of the AMI model over other factors (**Figure 8M**).

Similarly, the ICM diagnostic model showed high diagnostic accuracy with a risk score AUC of 0.996, and key genes *MRPS5* (AUC 0.929), *HNRNPA2B1* (AUC 0.877), and *METAP1* (AUC 0.855) also demonstrating high diagnostic accuracy (**Figures 8S–V**). The nomogram indicated that the expression of *MRPS5* notably enhances the diagnostic utility of the ICM model over other variables (**Figure 8W**). Calibration curve analysis and DCA(Decision Curve Analysis) showed that both AMI and ICM diagnostic models perform well with significant net benefits (**Figures 8K, L, X, Y**).

**Figure 9:**
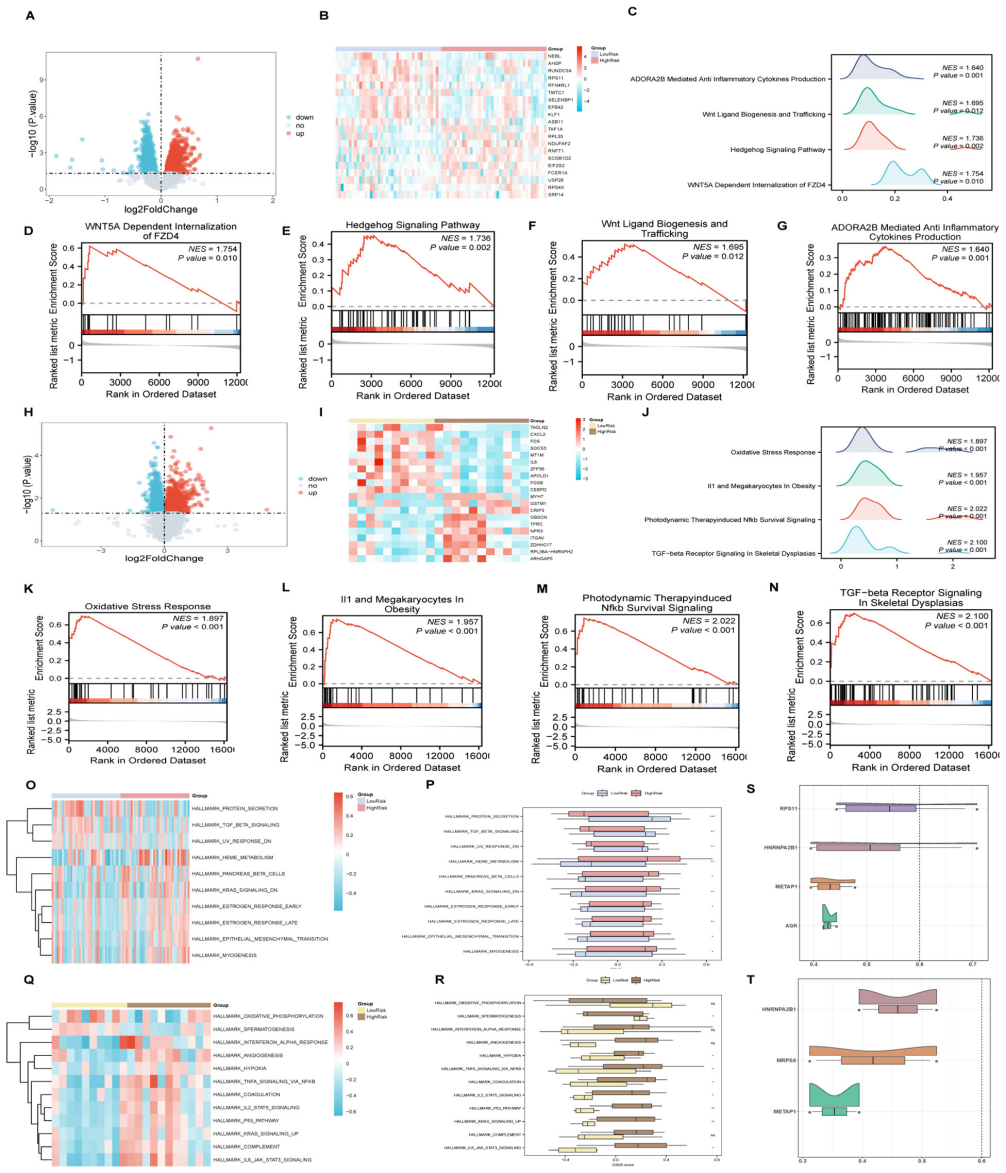
GSEA and GSVA for risk groups, friends analysis of AMI&ICM key genes. **(A-G)** GSEA for AMI high and low-risk groups, including volcano plots **(A)**, heat maps **(B)**, biological function mountain map **(C)** and enrichment pathways **(D-G)**. **(H-N)** GSEA for ICM high and low-risk groups, including volcano plots **(H)**, heat maps **(I)**, biological function mountain map **(J)** and enrichment pathways **(K-N)**. **(O, P)** GSVA analysis of AMI high and low-risk groups, illustrated by heat maps **(O)** and group comparison **(P)** maps. **(Q, R)** GSVA analysis for ICM high and low-risk groups, with corresponding heat maps **(Q)** and comparison maps **(R)**. **(S, T)** Friends analysis of key genes in AMI **(S)** and ICM **(T)**. (***p < 0.001, **p < 0.01, *p < 0.05, ns p >0.05).

Analysis conducted via the Friends algorithm indicated that *RPS11* is the gene proximate to the critical threshold (cut-off value = 0.60) in the context of AMI as depicted in **Figure 9S**. Similarly, *HNRNPA2B1* emerges as the gene nearest to the critical threshold (cut-off value = 0.60) within ICM, as illustrated in **Figure 9T**. These findings suggest that each of these genes holds a significant role in the pathogenesis of AMI and ICM, respectively.

### 3.9 GSEA for HighRisk and LowRisk groups

An analysis within the AMI samples identified 2504 DEGs(differentially expressed genes), meeting the criteria of having an |logFC |> 0 and a p-value < 0.05 between high and low-risk groups. The detailed subgroup classification method can be found in section 2.10. Among these DEGs, 1193 genes were upregulated, while 1311 were downregulated (**Figures 9A, B**). Subsequent GSEA revealed significant enrichment across various biological functions and signaling pathways including WNT5A-dependent internalization of FZD4, the Hedgehog signaling pathway, Wnt ligand biogenesis and trafficking, and ADORA2B-mediated production of anti-inflammatory cytokines (**Figures 9C–G**). The specific results of this analysis are documented in **Supplementary Table S11**.

Similarly, for ICM samples, 1875 DEGs (differentially expressed genes) met the established criteria, with 803 genes upregulated and 1072 genes downregulated (**Figures 9H, I**). The GSEA for these samples indicated significant enrichment in pathways associated with Oxidative Stress Response, IL1 and Megakaryocytes In Obesity, Photodynamic Therapy-induced NFkb Survival Signaling, and TGF-beta Receptor Signaling In Skeletal Dysplasias (**Figures 9J–N**), with comprehensive details provided in **Supplementary Table S12**.

### 3.10 GSVA for HighRisk and LowRisk groups

For AMI samples, GSVA differentiated the top 10 positively and negatively enriched pathways between HighRisk and LowRisk groups based on p-values less than 0.05 and logFC rankings (**Figures 9O, P**, **Supplementary Table S13**). The detailed subgroup classification method can be found in section 2.10. Validation via the Mann-Whitney U test reaffirmed the statistical significance (p-value < 0.05) of 10 pathways between the HighRisk and LowRisk groups, including pathways such as Myogenesis, KRAS Signaling Down, Epithelial-Mesenchymal Transition, Pancreas Beta Cells, Heme Metabolism, UV Response Down, Estrogen Response (Late and Early), TGF-beta Signaling, and Protein Secretion.

The analysis of ICM samples highlighted 12 pathways showing statistically significant differences between HighRisk and LowRisk groups (**Figures 9Q, R**, **Supplementary Table S14**), including pathways involved in P53 Pathway, IL6_JAK_STAT3 Signaling, TNFA Signaling Via NFKB, Hypoxia, and Spermatogenesis, KRAS Signaling Up, IL2_STAT5 Signaling, Coagulation.

### 3.11 ssGSEA for HighRisk and LowRisk groups

**Figure 10:**
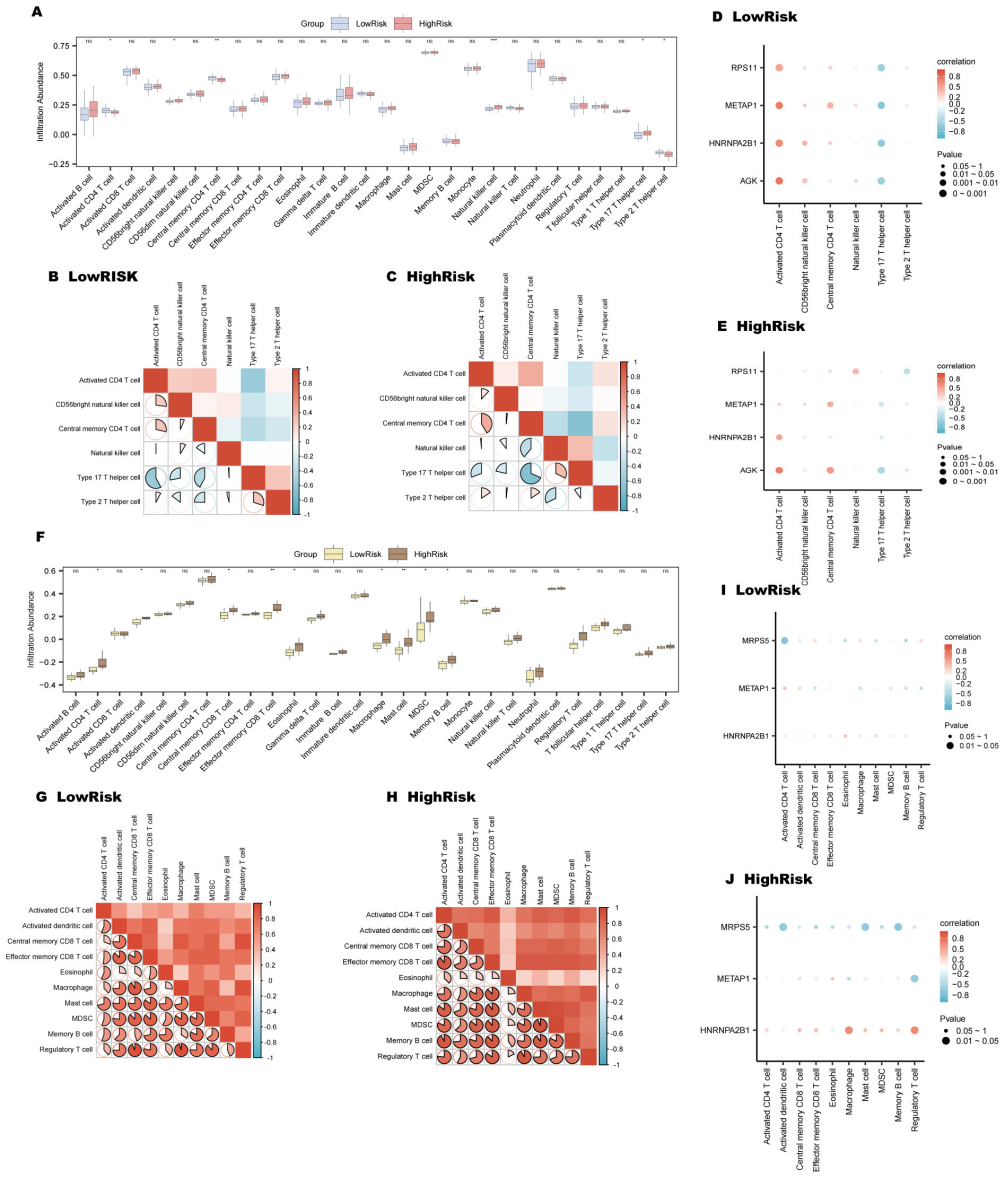
Immune infiltration analysis for AMI and ICM risk groups using ssGSEA. **(A-E)** AMI risk group immune infiltration analysis: Immune cell comparison in LowRisk and HighRisk groups **(A)**. Correlation of immune cell infiltration in HighRisk **(B)** and LowRisk **(C)** groups. Bubble plots of immune cell infiltration and Key Genes correlation in LowRisk **(D)** and HighRisk **(E)** groups. **(F-J)** ICM risk group immune infiltration analysis: Immune cell comparison in LowRisk and HighRisk groups **(F)**. Correlation of immune cell infiltration in LowRisk **(G)** and HighRisk **(H)** groups. Bubble plots of immune cell infiltration and Key Genes correlation in LowRisk **(I)** and HighRisk **(J)** groups. (ns, p-value ≥ 0.05, *p-value < 0.05, **p-value < 0.01, ***p < 0.001).

In AMI samples, ssGSEA analysis revealed distinct variations in the presence of six types of immune cells between the high and low-risk groups. Please refer to Section 2.10 for the detailed grouping method of high and low-risk subgroups. These included activated CD4+ T cells, CD56 bright natural killer cells, central memory CD4+ T cells, natural killer cells, Type 17 T helper cells, and Type 2 T helper cells, as illustrated in **Figure 10A**. Notably, the LowRisk group exhibited a substantial negative correlation between Type 17 T helper cells and activated CD4+ T cells(r-value = -0.579, p-value < 0.05) (**Figure 10B**), and similar trends were observed in the HighRisk group (**Figure 10C**). The gene *AGK* exhibited a significant positive association with activated CD4+ T cells in both risk groups, as shown in **Figures 10D, E**. Furthermore, the gene *RPS11* shows a positive correlation with natural killer cells in the HighRisk group (**Figure 10E**).

For ICM samples, ssGSEA indicated significant variations in 10 types of immune cells, including activated CD4+ T cells, central memory CD8+ T cells, effector memory CD8+ T cells, activated dendritic cells, eosinophils, macrophages, memory B cells, mast cells, myeloid-derived suppressor cells (MDSCs), and regulatory T cells (**Figure 10F**). In the LowRisk group, regulatory T cells and macrophages demonstrated a marked positive association, with an r-value of 0.952 and a p-value less than 0.05 (**Figure 10G**); while in the HighRisk group, mast cells and MDSCs displayed a significant positive correlation (r-value = 0.936, p-value < 0.05) (**Figure 10H**). Moreover, the gene *MRPS5* showed a notable negative association with activated CD4+ T cells in the LowRisk group (**Figure 10I**) and with memory B cells in the HighRisk group (**Figure 10J**).

### 3.12 Validation of findings via clinical samples and experiments

**Figure 11:**
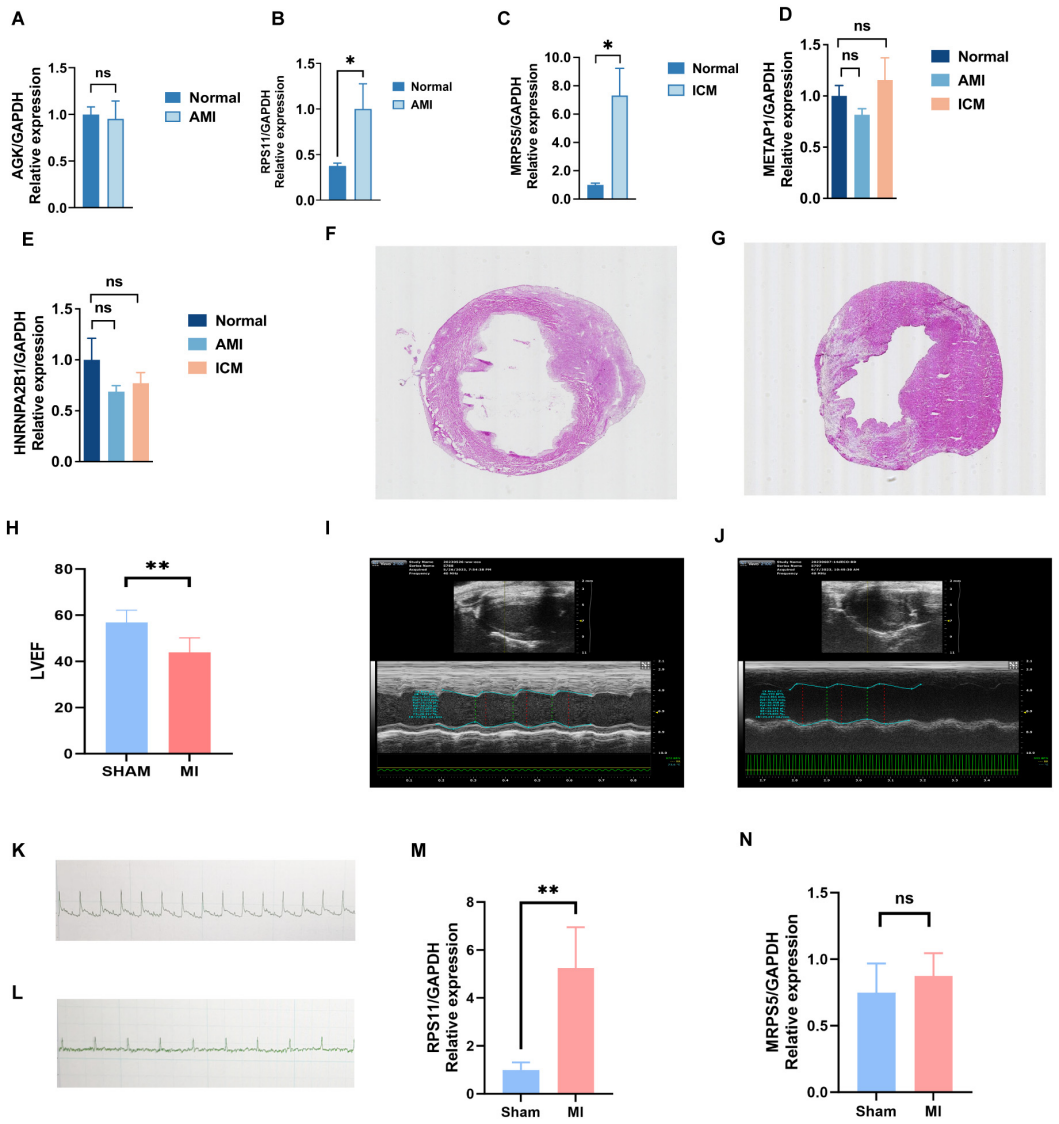
Validation results of experiment. **(A-E)** Key genes validated in peripheral blood from three groups using RT-PCR. **(F-N)** Validation of key genes in mice with myocardial infarction: electrocardiogram **(K, L)**, cardiac ultrasound **(I, J)**, HE staining **(F, G)**, LVEF **(H)**, and differences in the expression of mRNA levels of key genes, RPS11 **(M)** and MRP5 **(N)**, between the MI and Sham groups. (**p < 0.01, *p < 0.05, ns p >0.05).

Key genes were validated in peripheral blood from three groups using RT-PCR. The results showed a significant statistical difference in *RPS11* in the AMI group (p<0.05) and a significant statistical difference in *MRPS5* in the ICM group (p<0.05) (**Figures 11A–E**).This suggests that different mitophagy genes are involved and play distinct roles in the AMI phase and the chronic phase of ICM. During the acute phase, the increased expression of *RPS11* indicates its potential as a valuable diagnostic marker.

**Figure 12:**
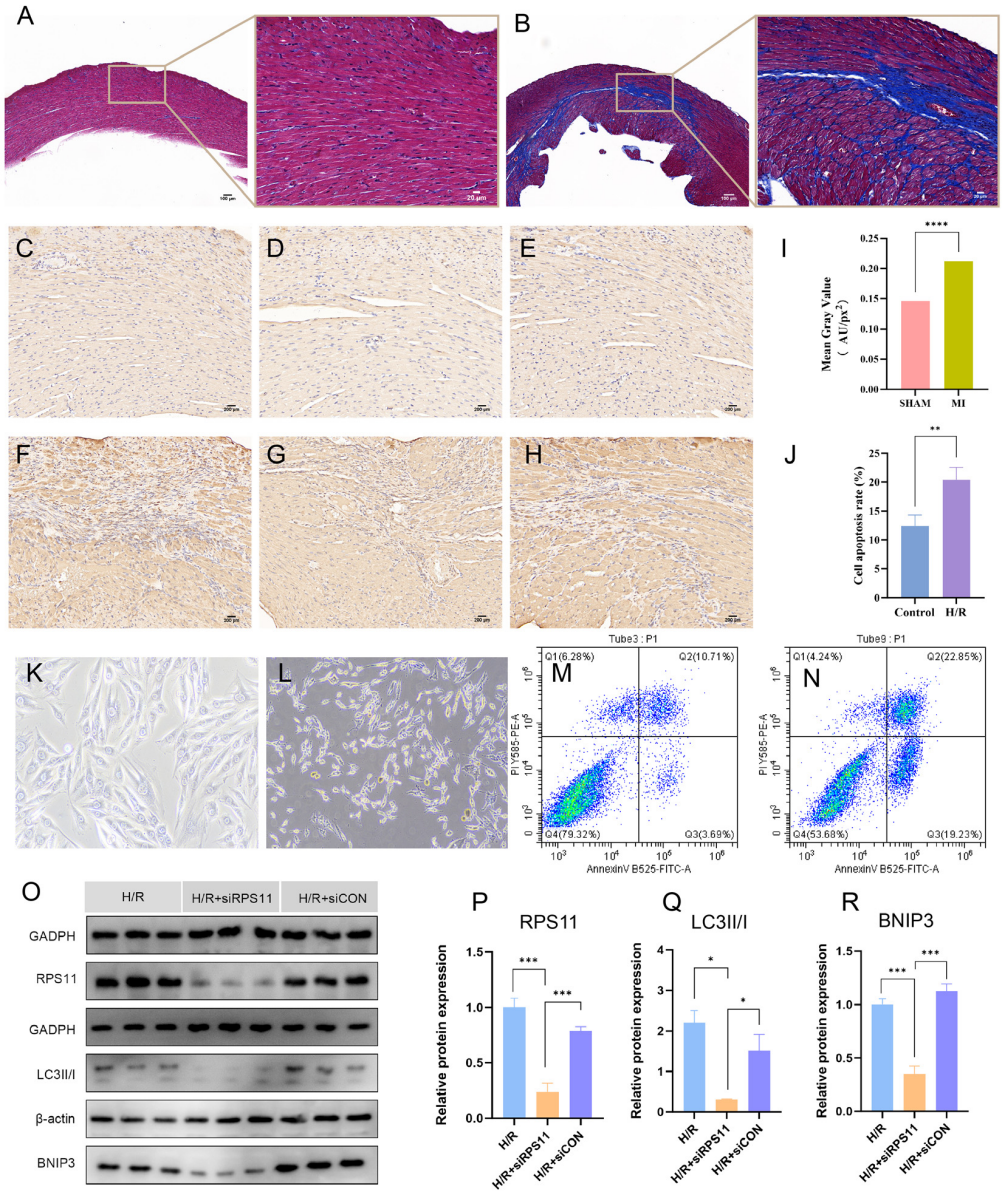
Validation results of experiment. **(A, B)** Masson’s trichrome staining results of the Sham group **(A)** and the MI group **(B)**. Fibrous tissue formation is observed in the infarct region of the MI group. **(C-H)** Immunohistochemical staining of *RPS11* protein in the Sham group **(C-E)** and MI group **(F-H)**. Notable brown *RPS11* protein expression is observed in the MI group. **(I)** Comparison of relative *RPS11* protein expression between the two groups in the immunohistochemical staining. **(K, L)** Light microscopy images of H9c2 cell morphology in the normoxic Control group **(K)** and hypoxia/reoxygenation (H/R) group **(L)**. **(J, M, N)** Flow cytometry analysis of cell apoptosis in the normoxic Control group **(M)** and H/R group **(N)**; **(J)** bar graph comparing apoptosis rates between the two groups. **(O-R)** Protein expression levels of *RPS11*, *LC3II/I*, and *BNIP3* in the H/R group, H/R + *RPS11* knockdown group (H/R+si*RPS11* group), and H/R + empty vector control group(H/R+siCON group). Western blot images **(O)**, and comparison of relative protein expression among the three groups: **(P)** *RPS11*, **(Q)** *LC3II/I*, and **(R)** *BNIP3*. (****p < 0.0001, ***p < 0.001, **p < 0.01, *p < 0.05).

To further investigate their effect on MI (Myocardial Infarction), we induced MI mice and performed Electrocardiographic (ECG) monitoring (**Figures 11K, L**) and cardiac ultrasonography (**Figures 11H–J**) to assess cardiac function 48 hours after MI. Electrocardiogram results confirmed the successful establishment of the acute myocardial infarction model. Ultrasonography results showed a significant decline in heart function following acute myocardial infarction, consistent with the heart failure criteria of ischemic cardiomyopathy. Mice were raised until 28 days of post-ligation, at which point cardiac tissue was collected. At 28 days (4 weeks), the myocardial tissue had already undergone the acute inflammatory phase, with increased fibrosis and the initiation of myocardial remodeling, marking a critical stage that impacts subsequent heart function. We performed hematoxylin and eosin (HE) staining (**Figures 11F, G**) on the heart tissue of the mice to visualize the area of infarction as well as the surrounding area. Additionally, further RT-PCR analyses (**Figures 11M, N**) were conducted, which revealed that the expression of *RPS11* was elevated in the infarcted mice group. The outcome of Masson staining (**Figures 12A, B**) showed that the myocardial tissue of the sham group was structurally intact, the cardiomyocytes were aligned and the collagen fibers in the interstitium were less stained and had a sporadic distribution, whereas the myocardial tissue of the infarct group was significantly damaged, with some areas of cardiomyocytes disorganized or broken, and the staining of collagen fibers in the fibrotic region was significantly increased (blue color).

Immunohistochemical staining of the animal model (**Figures 12C–I**) showed that the *RPS11* protein was mainly distributed in the cytoplasm of the cells, and the positive signals were brownish-yellow in color. The positive expression of *RPS11* in the infarct area was significantly increased in the MI group compared with the Sham group (p < 0.05). These findings suggest that *RPS11* exhibits significant differential expression not only during the acute phase of myocardial infarction (AMI) but also in the transition from the acute to the chronic phase.

Observation under the light microscope showed that the morphology of H9c2 cardiomyocytes in the normoxic control group was intact and evenly arranged, and the nuclei were clear, while the cells in the hypoxia-reoxygenation group (the H/R group) showed the damage characteristics of rounding, crumbling, widening of gaps, and partial rupture and detachment (as shown in **Figures 12K, L**), indicating that hypoxia-reoxygenation treatment had significantly affected the morphology of cardiomyocytes.

Cell flow assay results (**Figures 12M, N, J**) showed that the apoptosis rate of cardiomyocytes in the H/R group was significantly higher than that in the Control group (P < 0.05).

Western blot results (**Figures 12O–R**) showed that the expression of *RPS11, BNIP3*(BCL2 protein-interacting protein 3)*, and the LC3II/I*(Microtubule-associated protein 1 light chain 3II/I) ratio was significantly lower in the H/R+si*RPS11* group than in the H/R group (p < 0.05). The expression of the internal control protein as an internal reference protein was not significantly different in the two groups (p > 0.05). The expression levels of the individual proteins in the H/R+siCON group did not differ significantly from those of the H/R group (p > 0.05).The myocardial hypoxia-reoxygenation model simulates the cardiac status of acute myocardial infarction patients following emergency revascularization in real-world settings, providing strong evidence for a comprehensive understanding of the mechanisms underlying the transition from AMI to ICM.

## 4 Discussion

AMI represents a severe coronary artery disease with high mortality and disability rates. Recently, ICM as a complication of AMI has escalated, posing a significant public health issue. The transition from AMI to ICM is multifactorial, but the exact mechanisms are not fully understood. Mitophagy is crucial for cardiovascular homeostasis, removing damaged mitochondria to maintain cardiac integrity. Deficient mitophagy is linked to myocardial infarction and diabetic cardiomyopathy. Excessive mitophagy, however, may deplete mitochondria, impair ATP production, and trigger inflammation and apoptosis. Hence, mitophagy’s pathophysiological implications in AMI and ICM are crucial for developing targeted therapies.

In the BP annotations of GO and KEGG analyses, we found that these 11 hub MRDEGs were predominantly enriched in DNA biosynthesis processes and its regulation, telomere maintenance via elongation mechanisms, among other biological processes. The mitochondrion, a highly complex organelle, possesses its own genetic material, DNA polymerase, and RNA polymerase, thereby constituting an autonomous and comprehensive protein synthesis system, characterized by distinctive fusion and fission dynamics. Mitochondria are capable of repairing damage through genomic DNA repair mechanisms and several mitochondrial-specific DNA repair pathways (52).

Subsequent SVM and LASSO regression analyses identified four key genes associated with AMI (*RPS11, METAP1, HNRNPA2B1, AGK*) and three linked to ICM (*MRPS5, METAP1, HNRNPA2B1*). Diagnostic models based on these genes, validated using ROC curves, showed substantial diagnostic accuracy (AUCs of 0.83 and 0.996). ROC curve analyses indicated that *RPS11* had superior diagnostic utility among AMI genes (AUC 0.794), while *MRPS5* demonstrated the highest efficacy in the ICM cohort (AUC 0.929).

The gene *RPS11* encodes a member of the S17P family of the 40S ribosomal subunit, implicated in peptide chain elongation and mRNA activation subsequent to cap-complex and eukaryotic initiation factor (eIF) binding. While *RPS11* has not been previously reported in cardiac diseases, other ribosomal proteins(*RPS6)* have been associated with cardiac conditions (53). The mitochondrial ribosomal protein *MRPS5* represents a pivotal element of the mitochondrial translation mechanism. *MRPS5*’s functionality is intricately linked to cellular stress responses, wherein it disrupts mitochondrial structure and function by suppressing the expression of Klf15(Kruppel-like factor 15) through the l-phenylalanine/c-myc axis and the p-CREB/CREB(cAMP-response element binding protein) signaling pathway (54). Although research on these genes’ role in cardiovascular pathology is sparse, further investigation into their role in patient populations with AMI and ICM is imperative.

As two stages of disease, AMI and ICM exhibit both some similarities and distinct differences in terms of differentially expressed genes, activated signaling pathways, and immune cell involvement. Our analysis found that the TGF-β (Transforming Growth Factorβ) signaling pathway showed statistically significant differences in the GSVA analyses of AMI Combined Datasets, AMI HighRisk and LowRisk groups, and ICM HighRisk and LowRisk groups. This suggests that the TGF-β signaling pathway plays an important role in the regulation of acute myocardial infarction, the progression of ICM, and the transition from AMI to ICM.

The TGF-β (Transforming Growth Factorβ) signaling pathway relies on two main mechanisms, the Smad-dependent pathway and the non-Smad-dependent pathway, to achieve its biological functions. The role of TGF-β in Ischemic Cardiomyocytes remains controversial. In ischemia-reperfusion models, early administration of TGF-β1 has been shown to reduce cardiomyocyte apoptosis and infarct size through ERK1/2 (Extracellular signal-regulated kinase 1/2) activation (55). Vivo experiments using mice with cardiomyocyte-specific deletion of Tgfbr1(encoding TGF-β receptor 1, *ALK5*) or Tgfbr2(encoding TGF-β receptor 2) in non-reperfused MI models revealed that TGF-β signaling promotes left ventricular rupture by suppressing the transcription of genes encoding cardioprotective proteins such as IL-33(Interleukin-33), growth differentiation factor 15, and thrombospondin-4 (TSP4) (56). TGF-β also plays a key role in regulating inflammation, repair, and cardiac remodeling by inhibiting T helper 1 and cytotoxic T cell responses (57, 58) and inducing Treg cell differentiation. Meanwhile, TGF-β regulation of cardiac fibroblasts is critical for infarcted heart repair. Disruption of myofibroblast-specific SMAD3(Mothers Against Decapentaplegic Homolog 3) signaling impairs cardiac repair, inhibits integrin-mediated oxidative activity, and affects myofibroblast array formation, and SMAD3 deficiency is also associated with ventricular rupture and adverse remodeling (59). However, prolonged activation of TGF-β signaling may lead to increased fibrotic remodeling and diastolic dysfunction (60, 61). Therefore, dynamic regulation of the TGF-β pathway is required: early promotion of ECM (Extracellular Matrix) deposition via SMAD3 activation to prevent ventricular rupture and dilatation, and late inhibition of TGF-β-SMAD3 signaling to prevent excessive fibrosis and functional deterioration.

In the GSVA and GSEA analyses of ICM and its associated high- and low-risk groups, the TNFα(Tumor Necrosis Factor alpha)-mediated NF-κB (nuclear factor kappa-light-chain-enhancer of activated B cells) signaling pathway and Photodynamic Therapy-induced NF-κB Survival signaling pathway were significantly enriched. The role of NF-κB in myocardial infarction and subsequent heart failure is complex. Moderate activation of NF-κB has been shown to protect heart tissue by reducing cell damage and apoptosis after myocardial infarction (62). Conversely, studies report elevated NF-κB activity in the hearts of heart failure patients, which declines significantly after treatment with a left ventricular assist device, leading to improved cardiac function (63). Hypoxia further complicates this pathway by inducing hypoxia-inducible factor 1α, which activates NF-κB, promoting cardiomyocyte apoptosis (64). NF-κB also drives inflammation by enhancing the expression of *NLRP3*(NOD-like receptor family pyrin domain containing protein 3) and caspase-1, which facilitate the maturation of IL-1β(Interleukin-1β) and IL-18(Interleukin-18). This, in turn, triggers both local and systemic inflammatory responses. In addition, NF-κB regulates the expression of other inflammatory factors, such as IL-1β, which amplifies the inflammatory response and drives structural remodeling and fibrosis in the heart, exacerbating the condition (65, 66). Thus, long-term activation of the NF-κB signaling pathway and the TNFα pathway suggests the persistence of systemic and local inflammatory responses, which play an important role in the deterioration of cardiac function in ischemic cardiomyopathy.

This investigation employed ssGSEA within HighRisk and LowRisk AMI groups, unveiling no significant disparities in neutrophil infiltration between the groups, yet highlighting differences in various T cells and natural killer (NK) cell infiltrations. Prior studies have documented the migration of CD8+ and CD4+ T cell populations to the damaged myocardium during the cardiac repair phase (67). Consistent with our findings, Matsumoto et al. reported that NK cells facilitate myocardial cell death and exacerbate cardiac remodeling post-MI through the NKG2D/NKG2DL(Natural - Killer Group 2, Member D/Natural - Killer Group 2D Ligand) interaction (68). Furthermore, within the LowRisk group, activated CD4+ T cells exhibited positive correlations with *RPS11, METAP1, HNRNPA2B1*, and *AGK* genes, particularly with *AGK;* Type 17 T helper cells displayed negative correlations with these MRGs(mitophagy-related genes). T lymphocyte subgroups demonstrate considerable heterogeneity in T cell functionality, antigen recognition, and responsiveness to cardiac injury. Our analysis suggests potential associations between various T cells and mitophagy, revealing contrasting expression trends of *RPS11* with Type 17 T helper and with central memory CD4+ T cells in the LowRisk and HighRisk groups. In the high-risk group, *RPS11* shows a positive correlation with Natural Killer cells. *AGK* consistently exhibited a significant relationship with activated CD4+ T cells across both risk groups. The consistent and contrasting expression patterns of these MRGs(mitophagy-related genes) associated with T cells in different risk groups merit further investigation to elucidate potential pathophysiological implications.

The ssGSEA analysis of the ICM HighRisk and LowRisk groups revealed differences in the infiltration patterns of ten distinct immune cell types. In the LowRisk group, regulatory T cells and macrophages demonstrated the most significant positive correlation. Additionally, the gene *MRPS5* in the LowRisk group exhibited the most notable negative correlation with activated CD4+ T cells and, in the HighRisk group, the strongest negative correlation with memory B cells, Mast cells, Activated CD4+ T cells. Preliminary research into the depletion of B cell populations has indicated significant enhancements in myocardial recovery post-MI(Myocardial Infarction) (69). Nonetheless, the precise mechanisms or factors that orchestrate B cell activation in response to myocardial injury remain to be fully elucidated. This study is pioneering in proposing MRGs(mitophagy-related genes) associated with memory B cells, offering new insights into the mechanisms of B cell action in ICM.

This study constructed regulatory networks for the mRNA of hub MRDEGs with transcription factors (TFs), miRNAs, and RNA-binding proteins (RBPs) using multiple databases. The hub genes (*HNRNPA2B1, METAP1, XRN1, TNPO1*) were found to interact with twenty-seven miRNAs in the course of our study. miRNAs are capable of targeting mRNA to regulate key biological processes such as apoptosis, inflammation, fibrosis, and angiogenesis (70), thereby influencing the occurrence and progression of myocardial infarction (71, 72). Several miRNAs have been under consideration for their diagnostic capabilities and therapeutic applications (73). Elevated miR-1 (74)and miR-208 (75)are recognized as markers for the diagnosis of acute myocardial infarction (AMI). The role of miRNAs associated with mitophagy in diagnosis and prognosis is worth deeper exploration. Additionally, we constructed protein structures and integrated data from CTD databases to predict potential interactions with associated complexes. Valproic acid relates to *HNRNPA2B1*, *MRPS5*, and *GART*; acetaminophen to *MRPS5* and *TNPO1*; and cyclosporine to *HNRNPA2B1, LIG3, POLR2B*, and *TNPO1*. These compounds, widely used clinically, suggest potential roles in modulating mitophagy and treating AMI and ICM.

We confirmed our findings through clinical samples and experiments both in animals and cell models. First, blood samples from patients with acute myocardial infarction (AMI) and ischemic cardiomyopathy (ICM) showed clear differences. In AMI patients, *RPS11* levels were significantly higher, while *MRPS5* levels were notably different in ICM patients. 28 days after myocardial infarction, the acute inflammatory edema phase had ended, and the early scar formation phase began, marking the initiation of myocardial remodeling (76). Moreover, during this stage, the regulation of various neurohumoral mechanisms plays a role in affecting cardiac weight, fibrosis, and cardiac output. This is a critical period for the transition from AMI to ICM (77, 78). This time point provides more accurate information for the study of long-term pathological changes and potential therapeutic targets. Therefore, we established a 28-day myocardial infarction animal model to observe the expression of *RPS11*. The results showed that *RPS11* expression was not only upregulated at the mRNA level but also significantly increased in the infarcted area at the protein level, as revealed by immunohistochemistry. We then studied the role of *RPS11* in a hypoxia/reoxygenation (H/R) cell model using H9c2 cardiomyocytes. In the myocardial hypoxia/reoxygenation model, this acute injury process triggers a series of cellular responses and can effectively simulate the physiological changes observed in clinical patients with acute myocardial infarction who undergo revascularization (such as thrombolysis or coronary intervention).In this model, we reduced the expression of *RPS11* through gene knockdown and observed a subsequent decrease in the *LC3II/I* (Microtubule-associated protein 1 light chain 3 *II/I)*ratio and *BNIP3 (BCL2* protein-interacting protein 3) expression.

Microtubule-associated protein 1 light chain 3 (*LC3*), also called *Atg8*(Autophagy - related 8) in mammals, goes through several steps: modification, activation, and translocation (79). Ultimately *LC3* conjugates with the lipid phosphatidylethanolamine (79). After being lipidated, *LC3* serves as a linker or scaffold (80). It binds proteins containing LIR(*LC3* interaction region) motifs to the surfaces of the growing phagosome (81, 82). And the phagosome is released from the membrane (83). *LC3* is essential for extending the phagosome membrane, expanding the phagosome, and facilitating the fusion of autophagosomes with lysosomes (81, 84). The alteration in the *LC3II/I* ratio is closely associated with the initiation and progression of mitophagy.*BNIP3(BCL2* protein-interacting protein 3) is a protein located in the outer membrane of mitochondria (85). *BNIP3* is a member of the *Bcl-2* family (85).*BNIP3* is activated in response to stressful conditions (e.g., hypoxia, oxidative stress, etc.), and can directly contribute to mitophagy (86, 87). It can also recruit damaged or unwanted mitochondria into autophagic vesicles by interacting with autophagy-related proteins such as *LC3 (* 88). These results suggest that *RPS11* may affect mitophagy by regulating *BNIP3* levels. As part of the ribosome, Under conditions of oxidative stress, hypoxia, or other cellular damage, *RPS11* can regulate the expression of mitophagy pathway proteins, thereby promoting mitophagy to help maintain cellular homeostasis. However, prolonged and sustained overexpression of *RPS11* may lead to excessive mitophagy, resulting in excessive clearance of mitochondria, which can disrupt myocardial energy metabolism.

In our research, involving bioinformatics analyzing, clinical studying, and many experimental validations, we paid an attention to mitophagy and we presented the importance of *RPS11* in the event of acute myocardial infarction (AMI) and its transition to ischemic cardiomyopathy (ICM). Beginning our investigation with mitophagy has allowed us to delve deeper, but was also potentially responsible for the lack of discovery of novel biomarkers and pathways. These novel developments may be a groundwork for future progress. In future investigations, we will continue with an unrestricted attitude to uncover additional possibilities. We plan to conduct a more comprehensive analysis and explore additional relevant pathways to enhance the depth and breadth of our study. Due to the current limitations in our research conditions, many meaningful and yet-to-be-validated works remain unfinished, such as miRNAs related to mitophagy genes. We plan to conduct further clinical trials in the future to clarify their practical application value.

## 5 Conclusion

We investigated the regulatory mechanisms of mitophagy throughout AMI and ICM, identified key genes associated with these processes, and established accurate diagnostic models. The findings provide a significant foundation and novel insights for the diagnosis, risk stratification, and identification of potential therapeutic targets for AMI and ICM.

## Data Availability

The datasets analyzed for this study can be found in here: https://www.jianguoyun.com/p/DcnMDi0Q8uD-Cxi5p9QFIAA.

## Glossary

AMI: Acute Myocardial Infarction
ICM: Ischemic Cardiomyopathy
MI: Myocardial Infarction
DEGs: differentially expressed genes
MRGs: mitophagy-related genes
MRDEGs: mitophagy-related differentially expressed genes
PPI Network: Protein-protein Interaction Network
GO: Gene Ontology
KEGG: Kyoto Encyclopedia of Genes and Genomes enrichment analysis
GSEA: Gene Set Enrichment Analysis
GSVA: Gene Set Variation Analysis
TF: Transcription Factor
RBP: RNA-Binding Protein
SVM: Support Vector Machine
LASSO: Least Absolute Shrinkage and Selection Operator
ROC: Receiver Operating Characteristic
AUC: Area Under the Curve
DCA: Decision Curve Analysis
ssGSEA: single sample gene set enrichment analysis
I/R: ischemia-reperfusion
Drp1: dynamin - related protein 1
Mff: mitochondrial fission factor
ATP: Adenosine Triphosphate
H/R: Hypoxia/Reoxygenation
HE: Hematoxylin and Eosin
IHC: Immunohistochemistry
TGF-β: Transforming Growth Factorβ
Klf15: Kruppel-like factor 15
ERK1/2: Extracellular signal-regulated kinase ½
IL-33: Interleukin-33
CREB: cAMP-response element binding protein
SMAD3: Mothers Against Decapentaplegic Homolog 3
ECM: Extracellular Matrix
NF-κB: nuclear factor kappa-light-chain-enhancer of activated B cells
TNFα: Tumor Necrosis Factor alpha
*NLRP3*: NOD-like receptor family pyrin domain containing protein 3
IL-1β: Interleukin-1β
IL-18: Interleukin-18
NKG2D/NKG2DL: Natural - Killer Group 2, Member D/Natural - Killer Group 2D Ligand
BNIP3: BCL2 protein-interacting protein 3
LC3: Microtubule-associated protein 1 light chain 3
LIR: LC3 interaction region
PBS: Phosphate - Buffered Saline
DAB: 3,3’ Diaminobenzidine tetrahydrochloride
HRP: Horseradish Peroxidase
DMEM: Dulbecco’s Modified Eagle Medium
RIPA: Radioimmunoprecipitation Assay Buffer
BCA: Bicinchoninic Acid
SDS-PAGE: Sodium Dodecyl Sulfate - Polyacrylamide Gel Electrophoresis
PVDF: Polyvinylidene Fluoride
PBST: Phosphate - Buffered Saline Tween
Atg8: Autophagy - related 8
