# Supplementary material for: Identification and validation of mitophagy-related genes in acute myocardial infarction and ischemic cardiomyopathy and study of immune mechanisms across different risk groups
Source: Front Immunol. 2025 Mar 6;16:1486961. doi: 10.3389/fimmu.2025.1486961 (PMC11922711; doi:10.3389/fimmu.2025.1486961)
Supplement: Supplementary file 2 [file Table1.docx]

## Table 1 GEO Microarray Chip Information of AMI

|  | GSE48060 | GSE29532 |
| --- | --- | --- |
| Platform | GPL570 | GPL5175 |
| Type | Array | Array |
| Species | Homo sapiens | Homo sapiens |
| Tissue | Blood | Blood |
| Samples in AMI group | 31 | 49 |
| Samples in Control group | 21 | 6 |
| Reference | PMID: 24801707 | PMID: 23535507 |

GEO，Gene Expression Omnibus；AMI，Acute Myocardial Infarction。
