# Supplementary material for: Identification and validation of mitophagy-related genes in acute myocardial infarction and ischemic cardiomyopathy and study of immune mechanisms across different risk groups
Source: Front Immunol. 2025 Mar 6;16:1486961. doi: 10.3389/fimmu.2025.1486961 (PMC11922711; doi:10.3389/fimmu.2025.1486961)
Supplement: Supplementary file 3 [file Table2.docx]

## Table 2 GEO Microarray Chip Information of ICM

|  | GSE116250 | GSE46224 |
| --- | --- | --- |
| Platform | GPL16791 | GPL11154 |
| Type | High Throughput Sequencing | High Throughput Sequencing |
| Species | Homo sapiens | Homo sapiens |
| Tissue | Heart | Heart |
| Samples in ICM group | 13 | 8 |
| Samples in Control group | 14 | 8 |
| Reference | PMID: 30419824 | PMID: 24429688 |

GEO，Gene Expression Omnibus；ICM，Ischemic Cardiomyopathy。
