# Supplementary material for: Identification and validation of mitophagy-related genes in acute myocardial infarction and ischemic cardiomyopathy and study of immune mechanisms across different risk groups
Source: Front Immunol. 2025 Mar 6;16:1486961. doi: 10.3389/fimmu.2025.1486961 (PMC11922711; doi:10.3389/fimmu.2025.1486961)
Supplement: Supplementary file 5 [file Table4.docx]

**Table 3 Result of GO and KEGG Enrichment Analysis for Hub Genes**

| ONTOLOGY | ID | Description | GeneRatio | BgRatio | pvalue | p.adjust | qvalue |
| --- | --- | --- | --- | --- | --- | --- | --- |
| BP | GO:0071897 | DNA biosynthetic process | 3/11 | 198/18614 | 1.84E-04 | 1.84E-04 | 1.88E-02 |
| BP | GO:1904356 | regulation of telomere maintenance via telomere lengthening | 2/11 | 61/18614 | 5.70E-04 | 5.70E-04 | 2.91E-02 |
| BP | GO:0010833 | telomere maintenance via telomere lengthening | 2/11 | 81/18614 | 1.00E-03 | 1.00E-03 | 3.21E-02 |
| BP | GO:0032204 | regulation of telomere maintenance | 2/11 | 104/18614 | 1.65E-03 | 1.65E-03 | 3.21E-02 |
| BP | GO:2000278 | regulation of DNA biosynthetic process | 2/11 | 121/18614 | 2.22E-03 | 2.22E-03 | 3.21E-02 |
| CC | GO:0015935 | small ribosomal subunit | 2/11 | 74/19518 | 7.63E-04 | 7.63E-04 | 1.61E-02 |
| CC | GO:0000781 | chromosome, telomeric region | 2/11 | 169/19518 | 3.89E-03 | 3.89E-03 | 3.26E-02 |
| CC | GO:0044391 | ribosomal subunit | 2/11 | 185/19518 | 4.65E-03 | 4.65E-03 | 3.26E-02 |
| CC | GO:0005840 | ribosome | 2/11 | 229/19518 | 7.03E-03 | 7.03E-03 | 3.55E-02 |
| CC | GO:0005665 | RNA polymerase II, core complex | 1/11 | 15/19518 | 8.42E-03 | 8.42E-03 | 3.55E-02 |
| MF | GO:0016874 | ligase activity | 2/11 | 165/18369 | 4.18E-03 | 4.18E-03 | 1.67E-02 |
| MF | GO:0003735 | structural constituent of ribosome | 2/11 | 177/18369 | 4.80E-03 | 4.80E-03 | 1.67E-02 |
| MF | GO:0016742 | hydroxymethyl-, formyl- and related transferase activity | 1/11 | 10/18369 | 5.97E-03 | 5.97E-03 | 1.67E-02 |
| MF | GO:0051880 | G-quadruplex DNA binding | 1/11 | 10/18369 | 5.97E-03 | 5.97E-03 | 1.67E-02 |
| MF | GO:0098505 | G-rich strand telomeric DNA binding | 1/11 | 10/18369 | 5.97E-03 | 5.97E-03 | 1.67E-02 |
| KEGG | hsa03010 | Ribosome | 2/9 | 170/8661 | 1.26E-02 | 1.26E-02 | 8.24E-02 |
| KEGG | hsa00670 | One carbon pool by folate | 1/9 | 20/8661 | 2.06E-02 | 2.06E-02 | 8.24E-02 |
| KEGG | hsa01523 | Antifolate resistance | 1/9 | 30/8661 | 3.08E-02 | 3.08E-02 | 8.24E-02 |
| KEGG | hsa03020 | RNA polymerase | 1/9 | 34/8661 | 3.48E-02 | 3.48E-02 | 8.24E-02 |
| KEGG | hsa03410 | Base excision repair | 1/9 | 44/8661 | 4.48E-02 | 4.48E-02 | 8.49E-02 |

GO，Gene Ontology；BP，Biological Process；CC，Cellular Component；MF，Molecular Function；KEGG，Kyoto Encyclopedia of Genes and Genomes。
