# Supplementary material for: Identification and validation of mitophagy-related genes in acute myocardial infarction and ischemic cardiomyopathy and study of immune mechanisms across different risk groups
Source: Front Immunol. 2025 Mar 6;16:1486961. doi: 10.3389/fimmu.2025.1486961 (PMC11922711; doi:10.3389/fimmu.2025.1486961)
Supplement: Supplementary file 6 [file Table5.docx]

**Table 4 Results of GSEA for AMI Combined Datasets**

| ID | setSize | EnrichmentScore | NES | pvalue | p.adjust | qvalue |
| --- | --- | --- | --- | --- | --- | --- |
| REACTOME_NEUTROPHIL_DEGRANULATION | 355 | 0.50023 | 2.43001 | 1.00E-10 | 2.31E-07 | 2.15E-07 |
| REACTOME_IRAK4_DEFICIENCY_TLR2_4 | 16 | 0.87410 | 2.38978 | 2.35E-07 | 1.81E-04 | 1.68E-04 |
| REACTOME_REGULATION_OF_TLR_BY_ENDOGENOUS_LIGAND | 16 | 0.84410 | 2.30774 | 2.54E-06 | 9.76E-04 | 9.08E-04 |
| REACTOME_DISEASES_OF_IMMUNE_SYSTEM | 29 | 0.70617 | 2.28397 | 7.47E-06 | 2.15E-03 | 2.00E-03 |
| REACTOME_ANTIMICROBIAL_PEPTIDES | 46 | 0.62326 | 2.21013 | 8.98E-06 | 2.30E-03 | 2.14E-03 |
| REACTOME_TOLL_LIKE_RECEPTOR_CASCADES | 136 | 0.50251 | 2.16257 | 4.96E-08 | 5.72E-05 | 5.32E-05 |
| WP_MYD88_DISTINCT_INPUTOUTPUT_PATHWAY | 15 | 0.80453 | 2.15837 | 3.19E-05 | 5.25E-03 | 4.88E-03 |
| REACTOME_DEFENSINS | 14 | 0.79906 | 2.10364 | 1.53E-04 | 1.61E-02 | 1.50E-02 |
| REACTOME_TOLL_LIKE_RECEPTOR_TLR1_TLR2_CASCADE | 96 | 0.51851 | 2.07920 | 1.20E-06 | 6.94E-04 | 6.46E-04 |
| REACTOME_ROS_AND_RNS_PRODUCTION_IN_PHAGOCYTES | 29 | 0.63398 | 2.05048 | 3.41E-04 | 2.91E-02 | 2.71E-02 |
| NABA_ECM_AFFILIATED | 112 | 0.49105 | 2.04634 | 1.98E-06 | 9.11E-04 | 8.47E-04 |
| REACTOME_BETA_DEFENSINS | 11 | 0.81823 | 2.00258 | 1.93E-04 | 1.93E-02 | 1.80E-02 |
| PID_TOLL_ENDOGENOUS_PATHWAY | 22 | 0.65617 | 1.96171 | 1.16E-03 | 6.88E-02 | 6.41E-02 |
| REACTOME_TP53_REGULATES_TRANSCRIPTION_OF_CELL_DEATH_GENES | 37 | 0.57443 | 1.93660 | 1.16E-03 | 6.88E-02 | 6.41E-02 |
| KEGG_PANTOTHENATE_AND_COA_BIOSYNTHESIS | 13 | 0.74293 | 1.90644 | 1.35E-03 | 7.60E-02 | 7.07E-02 |
| REACTOME_TRANSFERRIN_ENDOCYTOSIS_AND_RECYCLING | 26 | 0.60704 | 1.90350 | 1.60E-03 | 8.60E-02 | 8.01E-02 |
| REACTOME_ACTIVATION_OF_IRF3_IRF7_MEDIATED_BY_TBK1_IKK_EPSILON | 14 | 0.71423 | 1.88030 | 4.73E-03 | 1.53E-01 | 1.43E-01 |
| WP_IL4_SIGNALING_PATHWAY | 48 | 0.49499 | 1.76947 | 1.95E-03 | 9.57E-02 | 8.91E-02 |
| BIOCARTA_IL1R_PATHWAY | 27 | 0.55020 | 1.75373 | 6.78E-03 | 1.66E-01 | 1.55E-01 |
| WP_SIGNAL_TRANSDUCTION_THROUGH_IL1R | 29 | 0.53527 | 1.73122 | 8.28E-03 | 1.75E-01 | 1.63E-01 |

GSEA，Gene Set Enrichment Analysis；AMI，Acute Myocardial Infarction。
