# Supplementary material for: Identification and validation of mitophagy-related genes in acute myocardial infarction and ischemic cardiomyopathy and study of immune mechanisms across different risk groups
Source: Front Immunol. 2025 Mar 6;16:1486961. doi: 10.3389/fimmu.2025.1486961 (PMC11922711; doi:10.3389/fimmu.2025.1486961)
Supplement: Supplementary file 7 [file Table6.docx]

**Table 5 Results of GSEA for ICM Combined Datasets**

| ID | setSize | EnrichmentScore | NES | pvalue | p.adjust | qvalue |
| --- | --- | --- | --- | --- | --- | --- |
| NABA_CORE_MATRISOME | 251 | 0.71507 | 2.76631 | 1.00E-10 | 4.94E-08 | 4.30E-08 |
| NABA_ECM_GLYCOPROTEINS | 173 | 0.69152 | 2.54802 | 1.00E-10 | 4.94E-08 | 4.30E-08 |
| PID_AP1_PATHWAY | 66 | 0.77051 | 2.47932 | 1.00E-10 | 4.94E-08 | 4.30E-08 |
| REACTOME_ECM_PROTEOGLYCANS | 76 | 0.71402 | 2.36845 | 2.57E-10 | 1.06E-07 | 9.21E-08 |
| REACTOME_NGF_STIMULATED_TRANSCRIPTION | 39 | 0.79370 | 2.32928 | 1.20E-08 | 2.69E-06 | 2.34E-06 |
| WP_OVERVIEW_OF_PROINFLAMMATORY_AND_PROFIBROTIC_MEDIATORS | 96 | 0.67637 | 2.31554 | 4.48E-10 | 1.38E-07 | 1.20E-07 |
| REACTOME_EXTRACELLULAR_MATRIX_ORGANIZATION | 282 | 0.58756 | 2.30441 | 1.00E-10 | 4.94E-08 | 4.30E-08 |
| NABA_PROTEOGLYCANS | 35 | 0.79785 | 2.29741 | 2.30E-08 | 4.73E-06 | 4.11E-06 |
| KEGG_GRAFT_VERSUS_HOST_DISEASE | 36 | 0.78006 | 2.26072 | 9.76E-08 | 1.21E-05 | 1.05E-05 |
| PID_FRA_PATHWAY | 36 | 0.77990 | 2.26027 | 9.76E-08 | 1.21E-05 | 1.05E-05 |
| REACTOME_DISEASES_ASSOCIATED_WITH_GLYCOSAMINOGLYCAN_METABOLISM | 41 | 0.76063 | 2.24599 | 1.13E-07 | 1.33E-05 | 1.16E-05 |
| PID_SYNDECAN_1_PATHWAY | 46 | 0.73871 | 2.23181 | 3.08E-07 | 2.87E-05 | 2.49E-05 |
| REACTOME_COLLAGEN_FORMATION | 81 | 0.65631 | 2.20487 | 5.61E-08 | 9.90E-06 | 8.61E-06 |
| REACTOME_ASSEMBLY_OF_COLLAGEN_FIBRILS_AND_OTHER_MULTIMERIC_STRUCTURES | 58 | 0.70330 | 2.20304 | 2.06E-07 | 2.22E-05 | 1.93E-05 |
| REACTOME_INTEGRIN_CELL_SURFACE_INTERACTIONS | 81 | 0.65442 | 2.19854 | 6.36E-08 | 1.05E-05 | 9.11E-06 |
| REACTOME_COLLAGEN_BIOSYNTHESIS_AND_MODIFYING_ENZYMES | 61 | 0.69081 | 2.18725 | 9.20E-08 | 1.21E-05 | 1.05E-05 |
| WP_INFLAMMATORY_RESPONSE_PATHWAY | 29 | 0.73063 | 2.02560 | 5.73E-05 | 2.44E-03 | 2.12E-03 |
| PID_IL12_2PATHWAY | 61 | 0.62918 | 1.99210 | 1.83E-05 | 9.60E-04 | 8.35E-04 |
| KEGG_TGF_BETA_SIGNALING_PATHWAY | 85 | 0.55530 | 1.85982 | 4.63E-05 | 2.08E-03 | 1.81E-03 |
| WP_PHOTODYNAMIC_THERAPYINDUCED_NFKB_SURVIVAL_SIGNALING | 32 | 0.64956 | 1.82743 | 1.13E-03 | 2.38E-02 | 2.07E-02 |

GSEA，Gene Set Enrichment Analysis；ICM，Ischemic Cardiomyopathy。
