# Supplementary material for: Identification and validation of mitophagy-related genes in acute myocardial infarction and ischemic cardiomyopathy and study of immune mechanisms across different risk groups
Source: Front Immunol. 2025 Mar 6;16:1486961. doi: 10.3389/fimmu.2025.1486961 (PMC11922711; doi:10.3389/fimmu.2025.1486961)
Supplement: Supplementary file 8 [file Table7.docx]

**Table 6 Results of GSVA for AMI Combined Datasets**

| ID | logFC | AveExpr | t | P.Value | adj.P.Val | B |
| --- | --- | --- | --- | --- | --- | --- |
| HALLMARK_TGF_BETA_SIGNALING | 0.16505 | 0.009716 | 3.115714 | 0.002285 | 0.114239 | -1.54266 |
| HALLMARK_CHOLESTEROL_HOMEOSTASIS | -0.13413 | -0.00273 | -2.45923 | 0.015316 | 0.263121 | -3.13452 |
| HALLMARK_REACTIVE_OXYGEN_SPECIES_PATHWAY | -0.1424 | 0.002013 | -2.44778 | 0.015787 | 0.263121 | -3.15932 |

GSVA，Gene Set Variation Analysis；AMI，Acute Myocardial Infarction。
