# Supplementary material for: Identification and validation of mitophagy-related genes in acute myocardial infarction and ischemic cardiomyopathy and study of immune mechanisms across different risk groups
Source: Front Immunol. 2025 Mar 6;16:1486961. doi: 10.3389/fimmu.2025.1486961 (PMC11922711; doi:10.3389/fimmu.2025.1486961)
Supplement: Supplementary file 9 [file Table8.docx]

**Table 7 Results of GSVA for ICM Combined Datasets**

| ID | logFC | AveExpr | t | P.Value | adj.P.Val | B |
| --- | --- | --- | --- | --- | --- | --- |
| HALLMARK_SPERMATOGENESIS | 0.325209 | 0.038138 | 6.384646 | 1.78E-08 | 4.45E-07 | 8.853881 |
| HALLMARK_E2F_TARGETS | 0.24209 | -0.0226 | 3.516224 | 0.000784 | 0.002449 | -1.51975 |
| HALLMARK_PEROXISOME | 0.176293 | -0.00872 | 2.749257 | 0.007641 | 0.018499 | -3.63535 |
| HALLMARK_HEME_METABOLISM | 0.158466 | 0.01965 | 2.743181 | 0.007769 | 0.018499 | -3.65051 |
| HALLMARK_UNFOLDED_PROTEIN_RESPONSE | -0.17327 | -0.01741 | -2.33521 | 0.022486 | 0.043243 | -4.6051 |
| HALLMARK_KRAS_SIGNALING_DN | -0.17553 | 0.014203 | -3.16138 | 0.002344 | 0.006895 | -2.54656 |
| HALLMARK_KRAS_SIGNALING_UP | -0.18515 | -0.01066 | -2.59406 | 0.011603 | 0.025224 | -4.01408 |
| HALLMARK_HEDGEHOG_SIGNALING | -0.19729 | -0.031 | -2.35654 | 0.02133 | 0.042659 | -4.55838 |
| HALLMARK_ALLOGRAFT_REJECTION | -0.21007 | -0.03201 | -2.37107 | 0.020572 | 0.042659 | -4.52633 |
| HALLMARK_GLYCOLYSIS | -0.21073 | 0.015862 | -3.68903 | 0.000449 | 0.001726 | -0.99229 |
| HALLMARK_CHOLESTEROL_HOMEOSTASIS | -0.2662 | -0.03425 | -3.69233 | 0.000444 | 0.001726 | -0.98206 |
| HALLMARK_COAGULATION | -0.29494 | -0.01842 | -4.25023 | 6.65E-05 | 0.000369 | 0.831027 |
| HALLMARK_MYOGENESIS | -0.29904 | -0.00494 | -5.17151 | 2.22E-06 | 2.22E-05 | 4.125627 |
| HALLMARK_APOPTOSIS | -0.30055 | -0.02774 | -3.94606 | 0.000191 | 0.000953 | -0.17719 |
| HALLMARK_P53_PATHWAY | -0.31684 | -0.01618 | -4.7172 | 1.23E-05 | 8.78E-05 | 2.460214 |
| HALLMARK_TNFA_SIGNALING_VIA_NFKB | -0.3723 | -0.02945 | -4.3101 | 5.38E-05 | 0.000336 | 1.034655 |
| HALLMARK_ANGIOGENESIS | -0.3841 | -0.0132 | -4.78056 | 9.72E-06 | 8.10E-05 | 2.688059 |
| HALLMARK_TGF_BETA_SIGNALING | -0.43575 | -0.02604 | -5.9291 | 1.13E-07 | 1.41E-06 | 7.039977 |
| HALLMARK_EPITHELIAL_MESENCHYMAL_TRANSITION | -0.47932 | -0.01893 | -6.19154 | 3.90E-08 | 6.51E-07 | 8.080406 |
| HALLMARK_NOTCH_SIGNALING | -0.54823 | -0.00117 | -9.67356 | 2.05E-14 | 1.03E-12 | 22.38816 |

GSVA，Gene Set Variation Analysis；ICM，Ischemic Cardiomyopathy。
