# Supplementary material for: Identification and validation of mitophagy-related genes in acute myocardial infarction and ischemic cardiomyopathy and study of immune mechanisms across different risk groups
Source: Front Immunol. 2025 Mar 6;16:1486961. doi: 10.3389/fimmu.2025.1486961 (PMC11922711; doi:10.3389/fimmu.2025.1486961)
Supplement: Supplementary file 10 [file Table9.docx]

**Table 8 Results of GSEA for AMI Risk Group**

| ID | setSize | EnrichmentScore | NES | pvalue | p.adjust | qvalue |
| --- | --- | --- | --- | --- | --- | --- |
| WP_HEMATOPOIETIC_STEM_CELL_DIFFERENTIATION | 47 | 0.58028 | 2.20931 | 5.15E-06 | 6.59E-04 | 5.77E-04 |
| REACTOME_CLASS_B_2_SECRETIN_FAMILY_RECEPTORS | 79 | 0.51010 | 2.16338 | 1.61E-06 | 3.70E-04 | 3.24E-04 |
| BIOCARTA_AHSP_PATHWAY | 10 | 0.86019 | 2.14395 | 1.19E-05 | 1.45E-03 | 1.26E-03 |
| REACTOME_CLASS_A_1_RHODOPSIN_LIKE_RECEPTORS | 262 | 0.41141 | 2.09186 | 2.33E-10 | 1.79E-07 | 1.57E-07 |
| REACTOME_GPCR_LIGAND_BINDING | 368 | 0.39382 | 2.08162 | 1.00E-10 | 1.15E-07 | 1.01E-07 |
| KEGG_NEUROACTIVE_LIGAND_RECEPTOR_INTERACTION | 233 | 0.40297 | 2.01194 | 3.18E-09 | 1.83E-06 | 1.60E-06 |
| REACTOME_ERYTHROCYTES_TAKE_UP_CARBON_DIOXIDE_AND_RELEASE_OXYGEN | 10 | 0.80715 | 2.01176 | 1.64E-04 | 9.23E-03 | 8.07E-03 |
| WP_OXIDATIVE_PHOSPHORYLATION | 28 | 0.58149 | 1.97669 | 4.97E-04 | 1.98E-02 | 1.73E-02 |
| WP_FOXA2_PATHWAY | 16 | 0.68689 | 1.97012 | 3.50E-04 | 1.60E-02 | 1.40E-02 |
| REACTOME_FOXO_MEDIATED_TRANSCRIPTION_OF_CELL_DEATH_GENES | 11 | 0.75505 | 1.94395 | 7.16E-04 | 2.70E-02 | 2.37E-02 |
| KEGG_PORPHYRIN_AND_CHLOROPHYLL_METABOLISM | 20 | 0.62763 | 1.92813 | 1.27E-03 | 3.57E-02 | 3.12E-02 |
| NABA_COLLAGENS | 37 | 0.53132 | 1.92001 | 8.39E-04 | 2.93E-02 | 2.56E-02 |
| REACTOME_COLLAGEN_CHAIN_TRIMERIZATION | 37 | 0.53132 | 1.92001 | 8.39E-04 | 2.93E-02 | 2.56E-02 |
| REACTOME_PHASE_4_RESTING_MEMBRANE_POTENTIAL | 16 | 0.66185 | 1.89832 | 1.10E-03 | 3.46E-02 | 3.03E-02 |
| WP_SMALL_LIGAND_GPCRS | 17 | 0.64674 | 1.89601 | 1.53E-03 | 3.96E-02 | 3.46E-02 |
| REACTOME_IMMUNOREGULATORY_INTERACTIONS_BETWEEN_A_LYMPHOID_AND_A_NON_LYMPHOID_CELL | 86 | 0.44223 | 1.89509 | 3.93E-05 | 3.49E-03 | 3.05E-03 |
| REACTOME_WNT5A_DEPENDENT_INTERNALIZATION_OF_FZD4 | 15 | 0.62132 | 1.75429 | 9.73E-03 | 1.19E-01 | 1.04E-01 |
| KEGG_HEDGEHOG_SIGNALING_PATHWAY | 48 | 0.45672 | 1.73630 | 2.28E-03 | 5.11E-02 | 4.47E-02 |
| REACTOME_WNT_LIGAND_BIOGENESIS_AND_TRAFFICKING | 24 | 0.52041 | 1.69500 | 1.17E-02 | 1.29E-01 | 1.13E-01 |
| REACTOME_ADORA2B_MEDIATED_ANTI_INFLAMMATORY_CYTOKINES_PRODUCTION | 107 | 0.36887 | 1.64026 | 1.01E-03 | 3.32E-02 | 2.91E-02 |

GSEA，Gene Set Enrichment Analysis；AMI，Acute Myocardial Infarction。
