# Supplementary material for: Identification and validation of mitophagy-related genes in acute myocardial infarction and ischemic cardiomyopathy and study of immune mechanisms across different risk groups
Source: Front Immunol. 2025 Mar 6;16:1486961. doi: 10.3389/fimmu.2025.1486961 (PMC11922711; doi:10.3389/fimmu.2025.1486961)
Supplement: Supplementary file 11 [file Table10.docx]

**Table 9 Results of GSEA for ICM Risk Group**

| ID | setSize | EnrichmentScore | NES | pvalue | p.adjust | qvalue |
| --- | --- | --- | --- | --- | --- | --- |
| NABA_CORE_MATRISOME | 251 | 0.72007 | 2.62187 | 1.00E-10 | 1.45E-08 | 1.15E-08 |
| NABA_ECM_GLYCOPROTEINS | 173 | 0.72139 | 2.51568 | 1.00E-10 | 1.45E-08 | 1.15E-08 |
| KEGG_COMPLEMENT_AND_COAGULATION_CASCADES | 65 | 0.81080 | 2.49215 | 1.00E-10 | 1.45E-08 | 1.15E-08 |
| REACTOME_INTERLEUKIN_10_SIGNALING | 43 | 0.86894 | 2.48654 | 1.00E-10 | 1.45E-08 | 1.15E-08 |
| WP_COMPLEMENT_SYSTEM | 89 | 0.75592 | 2.43975 | 1.00E-10 | 1.45E-08 | 1.15E-08 |
| WP_BURN_WOUND_HEALING | 96 | 0.73000 | 2.37949 | 1.00E-10 | 1.45E-08 | 1.15E-08 |
| KEGG_SYSTEMIC_LUPUS_ERYTHEMATOSUS | 55 | 0.79438 | 2.37854 | 1.66E-10 | 2.14E-08 | 1.69E-08 |
| PID_AP1_PATHWAY | 66 | 0.76992 | 2.37262 | 1.73E-10 | 2.14E-08 | 1.69E-08 |
| REACTOME_INTERLEUKIN_4_AND_INTERLEUKIN_13_SIGNALING | 102 | 0.72038 | 2.36794 | 1.00E-10 | 1.45E-08 | 1.15E-08 |
| WP_OVERVIEW_OF_PROINFLAMMATORY_AND_PROFIBROTIC_MEDIATORS | 96 | 0.72354 | 2.35845 | 1.00E-10 | 1.45E-08 | 1.15E-08 |
| WP_SPINAL_CORD_INJURY | 110 | 0.71235 | 2.35482 | 1.00E-10 | 1.45E-08 | 1.15E-08 |
| WP_COMPLEMENT_AND_COAGULATION_CASCADES | 56 | 0.78972 | 2.34956 | 8.32E-10 | 8.94E-08 | 7.05E-08 |
| WP_ALLOGRAFT_REJECTION | 84 | 0.72802 | 2.34800 | 2.85E-10 | 3.35E-08 | 2.64E-08 |
| PID_FRA_PATHWAY | 36 | 0.83133 | 2.31356 | 8.21E-09 | 6.99E-07 | 5.51E-07 |
| WP_TYROBP_CAUSAL_NETWORK_IN_MICROGLIA | 58 | 0.76553 | 2.29316 | 1.52E-09 | 1.56E-07 | 1.23E-07 |
| REACTOME_EXTRACELLULAR_MATRIX_ORGANIZATION | 282 | 0.61851 | 2.28517 | 1.00E-10 | 1.45E-08 | 1.15E-08 |
| WP_TGFBETA_RECEPTOR_SIGNALING_IN_SKELETAL_DYSPLASIAS | 58 | 0.70092 | 2.09964 | 1.08E-06 | 5.34E-05 | 4.21E-05 |
| WP_PHOTODYNAMIC_THERAPYINDUCED_NFKB_SURVIVAL_SIGNALING | 32 | 0.74813 | 2.02240 | 3.71E-05 | 1.01E-03 | 7.94E-04 |
| WP_IL1_AND_MEGAKARYOCYTES_IN_OBESITY | 24 | 0.75838 | 1.95671 | 1.86E-04 | 3.82E-03 | 3.01E-03 |
| WP_OXIDATIVE_STRESS_RESPONSE | 32 | 0.70178 | 1.89709 | 3.91E-04 | 7.01E-03 | 5.53E-03 |

GSEA，Gene Set Enrichment Analysis；ICM，Ischemic Cardiomyopathy。
