# Supplementary material for: Identification and validation of mitophagy-related genes in acute myocardial infarction and ischemic cardiomyopathy and study of immune mechanisms across different risk groups
Source: Front Immunol. 2025 Mar 6;16:1486961. doi: 10.3389/fimmu.2025.1486961 (PMC11922711; doi:10.3389/fimmu.2025.1486961)
Supplement: Supplementary file 12 [file Table11.docx]

**Table 10 Results of GSVA for AMI Risk Group**

| ID | logFC | AveExpr | t | P.Value | adj.P.Val | B |
| --- | --- | --- | --- | --- | --- | --- |
| HALLMARK_PROTEIN_SECRETION | 0.234985 | 0.01875 | 3.82745 | 0.000228 | 0.01139 | 0.352351 |
| HALLMARK_TGF_BETA_SIGNALING | 0.16353 | 0.007639 | 3.16354 | 0.002074 | 0.034572 | -1.65401 |
| HALLMARK_UV_RESPONSE_DN | 0.123371 | -0.00309 | 2.692914 | 0.008329 | 0.060724 | -2.88809 |
| HALLMARK_EPITHELIAL_MESENCHYMAL_TRANSITION | -0.11682 | -0.0109 | -2.13505 | 0.035243 | 0.176217 | -4.12669 |
| HALLMARK_ESTROGEN_RESPONSE_EARLY | -0.13511 | -0.01361 | -2.68555 | 0.008501 | 0.060724 | -2.90607 |
| HALLMARK_MYOGENESIS | -0.14582 | -0.00139 | -2.42229 | 0.017255 | 0.095863 | -3.52055 |
| HALLMARK_KRAS_SIGNALING_DN | -0.15872 | -0.02457 | -2.51272 | 0.013608 | 0.085048 | -3.31571 |
| HALLMARK_PANCREAS_BETA_CELLS | -0.16723 | -0.01568 | -2.88808 | 0.004768 | 0.047677 | -2.39645 |
| HALLMARK_ESTROGEN_RESPONSE_LATE | -0.17002 | -0.00354 | -3.62508 | 0.00046 | 0.011496 | -0.29008 |
| HALLMARK_HEME_METABOLISM | -0.20826 | -0.00769 | -2.94368 | 0.004047 | 0.047677 | -2.2511 |
| HALLMARK_PROTEIN_SECRETION1 | 0.234985 | 0.01875 | 3.82745 | 0.000228 | 0.01139 | 0.352351 |
| HALLMARK_TGF_BETA_SIGNALING1 | 0.16353 | 0.007639 | 3.16354 | 0.002074 | 0.034572 | -1.65401 |
| HALLMARK_UV_RESPONSE_DN1 | 0.123371 | -0.00309 | 2.692914 | 0.008329 | 0.060724 | -2.88809 |
| HALLMARK_EPITHELIAL_MESENCHYMAL_TRANSITION1 | -0.11682 | -0.0109 | -2.13505 | 0.035243 | 0.176217 | -4.12669 |
| HALLMARK_ESTROGEN_RESPONSE_EARLY1 | -0.13511 | -0.01361 | -2.68555 | 0.008501 | 0.060724 | -2.90607 |
| HALLMARK_MYOGENESIS1 | -0.14582 | -0.00139 | -2.42229 | 0.017255 | 0.095863 | -3.52055 |
| HALLMARK_KRAS_SIGNALING_DN1 | -0.15872 | -0.02457 | -2.51272 | 0.013608 | 0.085048 | -3.31571 |
| HALLMARK_PANCREAS_BETA_CELLS1 | -0.16723 | -0.01568 | -2.88808 | 0.004768 | 0.047677 | -2.39645 |
| HALLMARK_ESTROGEN_RESPONSE_LATE1 | -0.17002 | -0.00354 | -3.62508 | 0.00046 | 0.011496 | -0.29008 |
| HALLMARK_HEME_METABOLISM1 | -0.20826 | -0.00769 | -2.94368 | 0.004047 | 0.047677 | -2.2511 |

GSVA，Gene Set Variation Analysis；AMI，Acute Myocardial Infarction。
