# Supplementary material for: Identification and validation of mitophagy-related genes in acute myocardial infarction and ischemic cardiomyopathy and study of immune mechanisms across different risk groups
Source: Front Immunol. 2025 Mar 6;16:1486961. doi: 10.3389/fimmu.2025.1486961 (PMC11922711; doi:10.3389/fimmu.2025.1486961)
Supplement: Supplementary file 13 [file Table12.docx]

**Table 11 Results of GSVA for ICM Risk Group**

| ID | logFC | AveExpr | t | P.Value | adj.P.Val | B |
| --- | --- | --- | --- | --- | --- | --- |
| HALLMARK_OXIDATIVE_PHOSPHORYLATION | 0.266798 | 0.03779 | 2.146141 | 0.035734 | 0.163966 | -3.80288 |
| HALLMARK_SPERMATOGENESIS | 0.213818 | 0.03116 | 2.104195 | 0.039372 | 0.164049 | -3.88052 |
| HALLMARK_HYPOXIA | -0.2246 | -0.00656 | -2.20438 | 0.031171 | 0.163966 | -3.69294 |
| HALLMARK_COMPLEMENT | -0.24975 | -0.00402 | -2.2797 | 0.026034 | 0.162712 | -3.54708 |
| HALLMARK_KRAS_SIGNALING_UP | -0.25941 | -0.01855 | -2.385 | 0.02011 | 0.162712 | -3.3363 |
| HALLMARK_IL2_STAT5_SIGNALING | -0.26515 | -0.03775 | -2.33266 | 0.022885 | 0.162712 | -3.44206 |
| HALLMARK_COAGULATION | -0.26554 | -0.01111 | -2.40665 | 0.019054 | 0.162712 | -3.29202 |
| HALLMARK_INTERFERON_ALPHA_RESPONSE | -0.29159 | -0.02871 | -2.14208 | 0.036072 | 0.163966 | -3.81045 |
| HALLMARK_ANGIOGENESIS | -0.31618 | -0.01249 | -2.73801 | 0.008032 | 0.120675 | -2.57423 |
| HALLMARK_TNFA_SIGNALING_VIA_NFKB | -0.32626 | -0.0072 | -2.66964 | 0.009654 | 0.120675 | -2.72829 |
| HALLMARK_HYPOXIA1 | -0.2246 | -0.00656 | -2.20438 | 0.031171 | 0.163966 | -3.69294 |
| HALLMARK_COMPLEMENT1 | -0.24975 | -0.00402 | -2.2797 | 0.026034 | 0.162712 | -3.54708 |
| HALLMARK_KRAS_SIGNALING_UP1 | -0.25941 | -0.01855 | -2.385 | 0.02011 | 0.162712 | -3.3363 |
| HALLMARK_IL2_STAT5_SIGNALING1 | -0.26515 | -0.03775 | -2.33266 | 0.022885 | 0.162712 | -3.44206 |
| HALLMARK_COAGULATION1 | -0.26554 | -0.01111 | -2.40665 | 0.019054 | 0.162712 | -3.29202 |
| HALLMARK_INTERFERON_ALPHA_RESPONSE1 | -0.29159 | -0.02871 | -2.14208 | 0.036072 | 0.163966 | -3.81045 |
| HALLMARK_ANGIOGENESIS1 | -0.31618 | -0.01249 | -2.73801 | 0.008032 | 0.120675 | -2.57423 |
| HALLMARK_TNFA_SIGNALING_VIA_NFKB1 | -0.32626 | -0.0072 | -2.66964 | 0.009654 | 0.120675 | -2.72829 |
| HALLMARK_P53_PATHWAY | -0.3419 | -0.03378 | -3.17163 | 0.002346 | 0.079476 | -1.53006 |
| HALLMARK_IL6_JAK_STAT3_SIGNALING | -0.3684 | -0.01505 | -3.068 | 0.003179 | 0.079476 | -1.78974 |

GSVA，Gene Set Variation Analysis；ICM，Ischemic Cardiomyopathy。
